# Features and advantages of flexible silicon nanowires for SERS applications

**DOI:** 10.3762/bjnano.10.72

**Published:** 2019-03-15

**Authors:** Hrvoje Gebavi, Vlatko Gašparić, Dubravko Risović, Nikola Baran, Paweł Henryk Albrycht, Mile Ivanda

**Affiliations:** 1 Ruđer Bošković Institute, Division of Materials Physics, Laboratory for Molecular Physics and Synthesis of New Materials, Bijenička cesta 54, Zagreb, Croatia; 2 Center of Excellence for Advanced Materials and Sensing Devices, Research Unit New Functional Materials, Bijenička cesta 54, Zagreb, Croatia; 3 Institute of Physical Chemistry, Polish Academy of Sciences, Kasprzaka 44/52, 01-224 Warsaw, Poland

**Keywords:** flexible hot spots, horizontal silicon nanowires, 4-mercaptophenylboronic acid, surface-enhanced Raman spectroscopy (SERS), vapour–liquid–solid

## Abstract

The paper reports on the features and advantages of horizontally oriented flexible silicon nanowires (SiNWs) substrates for surface-enhanced Raman spectroscopy (SERS) applications. The novel SERS substrates are described in detail considering three main aspects. First, the key synthesis parameters for the flexible nanostructure SERS substrates were optimized. It is shown that fabrication temperature and metal-plating duration significantly influence the flexibility of the SiNWs and, consequently, determine the SERS enhancement. Second, it is demonstrated how the immersion in a liquid followed by drying results in the formation of SiNWs bundles influencing the surface morphology. The morphology changes were described by fractal dimension and lacunar analyses and correlated with the duration of Ag plating and SERS measurements. SERS examination showed the optimal intensity values for SiNWs thickness values of 60–100 nm. That is, when the flexibility of the self-assembly SiNWs allowed hot spots occurrence. Finally, the test with 4-mercaptophenylboronic acid showed excellent SERS performance of the flexible, horizontally oriented SiNWs in comparison with several other commercially available substrates.

## Introduction

The mechanism of surface-enhanced Raman spectroscopy (SERS) [1] is predominantly described by electromagnetic theory, which covers most of the observed features [2]. Specially designed nanostructured surfaces, preferably with clusters of metal nanoparticles, sharp edges and tips, are the key to strong electromagnetic enhancement ranging from 10^10^ to 10^14^ [3]. If the values of Raman cross section of the analyte and of SERS enhancement are appropriate, even single-molecule detection is possible. For example, under resonant laser excitation of analyte molecules with differential cross section of ca. 10^−27^ cm^2^/sr, a SERS enhancement factor (EF) of 10^8^ would be adequate for single-molecule detection. Under non-resonant conditions and/or for lower cross sections (ca. 10^−30^ cm^2^/sr ) EF values above 10^11^ are required [4–5]. The possibility of detecting molecules at low concentrations leads to numerous applications in medicine [6], biology [7], gas [8] and chemical sensing [9], agriculture [10], food science [11–12]. Therefore, SERS is currently considered a hot topic in scientific research.

Generally, SERS-active nanostructures are used on either colloidal or solid substrates. A carefully prepared substrate for a specifically targeted molecule is of the crucial importance for the low SERS detection limit. The nanostructured surface significantly increases the effective SERS surface area of the substrates. Colloids are economical for synthesis, but suffer from the lack of reproducibility due to unpredictable aggregation. Thus, researchers have implemented various ways to control the aggregation, such as bifunctional linker molecules, stimuli-responsive polymers, short single-stranded DNA chains or aptamers. Optimized solid substrates offer high measurement reproducibility, stability, the possibility of precise spot-determined analyte detection and the measurement of water-insoluble substances [12].

Nowadays, the scientific focus is on a subcategory of solid substrates, i.e., “flexible SERS substrates”, which unlike the conventional solid substrates conform to the specific object and efficiently extract the target molecules [13–18]. They can withstand a tensile strain of up to 30% without losing the SERS features [13]. These flexible substrates include materials such as polydimethylsiloxane (PDMS) [13,15] or poly(methyl methacrylate) (PMMA) [14]. However, apart from the flexible substrates, also flexible nanostructures are reported on conventional, solid SERS substrates [19–21]. In these reports, vertically oriented silicon nanopillars in contact with a liquid would lean towards each other, trapping the targeted molecule. The Raman signal of these commercially available substrates exceeds that of competitors [19]. Therefore, these substrates can be considered as one of the top SERS substrates on the market.

We have synthesized similar flexible, but horizontally oriented silicon nanowires (SiNWs), and observed a significant increase of SERS intensity after immersion into the liquid. The surface tension of the liquid influences position and shape of the SiNWs. The SiNWs are displaced and pulled together in bundles. As a result, flexible hot spots with significantly increased SERS intensity occur. During the synthesis of flexible SiNWs the fabrication parameters are of a crucial importance. The small-diameter SiNWs synthesized and described in this paper are sensitive not only to the surface tension of the liquid. Their flexibility also depends on the metal plating [22]. The paper compares Ag-plated horizontally synthesized SiNWs with commercially available vertically aligned SiNWs for SERS applications utilizing 4-mercaptophenylboronic acid (4-MPBA) as a test molecule. To the best of our knowledge, flexible horizontally oriented SiNWs and the benefits of flexible hot spots for SERS have not been reported before.

We have compared the synthesized substrates (RBI) with commercially available substrates from Silmeco (https://www.silmeco.com), AtoID (http://atoid.com) and Sersitive (http://sersitive.eu). One should be aware that the presented results are obtained on only with a few commercially available substrates and that our intention is not to rate or evaluate, but rather the presentation of the first results.

## Experimental

Horizontal silicon nanowires were fabricated by vapor–liquid–solid (VLS) synthesis in a low-pressure chemical vapor deposition (LPCVD) reactor as described in [23]. In short, Si wafers (<100> orientation, 5–10 Ω·cm resistivity, p-type) were cleaned following the standard RCA (Radio Corporation of America) cleaning processes [24], followed by Au sputtering in a Polaron E5000 sputter coater at ca. 5·10^−4^ mbar work pressure. Prior to VLS synthesis, annealing in vacuum for one hour at temperatures from 480–560 °C was performed. In the VLS process, 26% SiH_4_ diluted in Ar with 270 sccm flow rate was deposited for 1 h. In each experiment, the annealing temperature was the same as the VLS process temperature. The Ag nanoparticle decoration of the horizontal SiNWs obtained in the previous step was obtained by the same sputtering system after different time durations (3, 5, 7, 16, 20 and 30 min). Afterwards, the ca. 3 × 3 mm^2^ squared samples were immersed in an ethanol solution of 4-mercaptophenylboronic acid (4-MPBA) for several hours.

The morphology of the synthesized samples was monitored with a Jeol JSM 7000F scanning electron microscope under 10 kV discharge.

Raman spectroscopy measurements were performed using a Jobin Yvon T64000 Raman spectrometer in micro-single configuration. The laser power at 532 nm on the sample in the ca. 1 μm spot was 1–2 mW. For all experiments, a long-working-distance 50×/0.75 objective was used. The exposition time was 10 or 20 s per scan.

For the determination of fractal dimension and lacunarity, we used the ImageJ software [25] with the FracLac plugin. The data were extracted from grey-scale images using ‘Box Counting – ‘Differential volume Plus1’ for grey-scale image analyses with “black background” as fixed option. The program operates with the equation: *D* = 3 − (*s*/2), where *s* is the regression-line slope, and for the average fractal dimension,


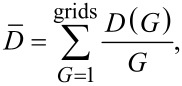


where the summation is over all grids.

## Results and Discussion

### Dependence of SERS intensity on the VLS process temperature

The first step in the synthesis optimization of the horizontally oriented SiNW substrates includes the determination of the optimal VLS synthesis temperature. The color of the substrates ranged from pale yellow to dark brown (Supporting Information File 1, Figure S1). The color change clearly indicates changes of thickness and morphology of the SiNWs induced by the processing temperature (Supporting Information File 1, Figure S2). Roughly, the SiNW diameter increases with VLS process temperature from 50 to 150 nm. Similar values and a linear correlation between temperature and thickness were reported in [26].

4-MPBA was chosen as a SERS test molecule because of the strong affinity between the thiol group and metal surfaces (Ag or Au) as well as because of the easy formation of self-assembled monolayers (SAMs) [27]. Furthermore, the benzene ring is orientation-sensitive and has a relatively large Raman cross section (ca. 10^−29^ cm^2^/sr [28]). The boronic acid group binds to certain analytes, for example, peptidoglycans in bacterial cell walls [29]. Recently, the difficult detection of saccharides (glucose, fructose) due to a low Raman scattering cross-section and a weak metal affinity was facilitated through the surface immobilization via 4-MPBA [30–31]. The 4-MPBA reporter features are predominantly based on re-orientation i.e. binding of the analyte via the boronic acid group causing a symmetry breaking and activation of the charge transfer mechanism which finally impacts SERS intensity [32].

It is also known that MPBA is pH-sensitive [30,33]. The bands at 1000 and 1073 cm^−1^ gradually decrease with increasimg pH value, which can be ascribed to the change of the angle between the S–H bond and the metal surface (Supporting Information File 1, Figure S3). With the increase of the pH value, the sp^2^-hybridized boronic acid changes to the sp^3^-hybridized boronate [33].

**Figure 1:**
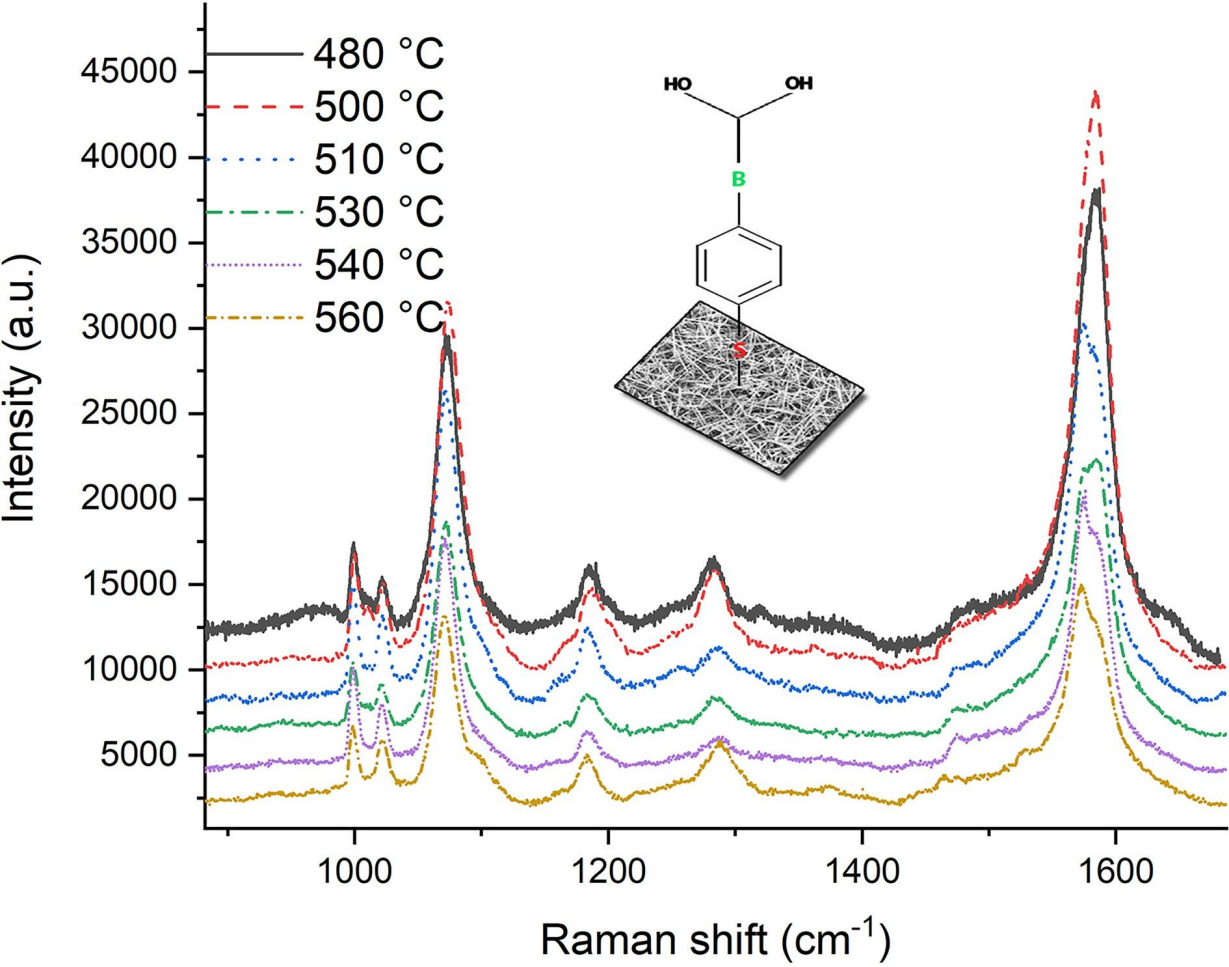
SERS spectra of 10^−4^ M 4-MPBA (inset) after immersion in H_2_O for different VLS process temperatures.

The fabricated horizontal SiNWs synthesized at different temperatures in the range from 480 to 560 °C were all sputtered with Ag for 5 min. After that, the samples were dipped in 10^−4^ M MPBA solution in ethanol for several hours in order to allow for the formation of SAMs. After the incubation, the samples were washed with milliQ water and dried for 1 h. SERS spectra are presented in Figure 1. The full-range 4-MPBA SERS spectrum is shown in Supporting Information File 1, Figure S4. The band assignments are given in Table S1 of Supporting Information File 1.

**Figure 2:**
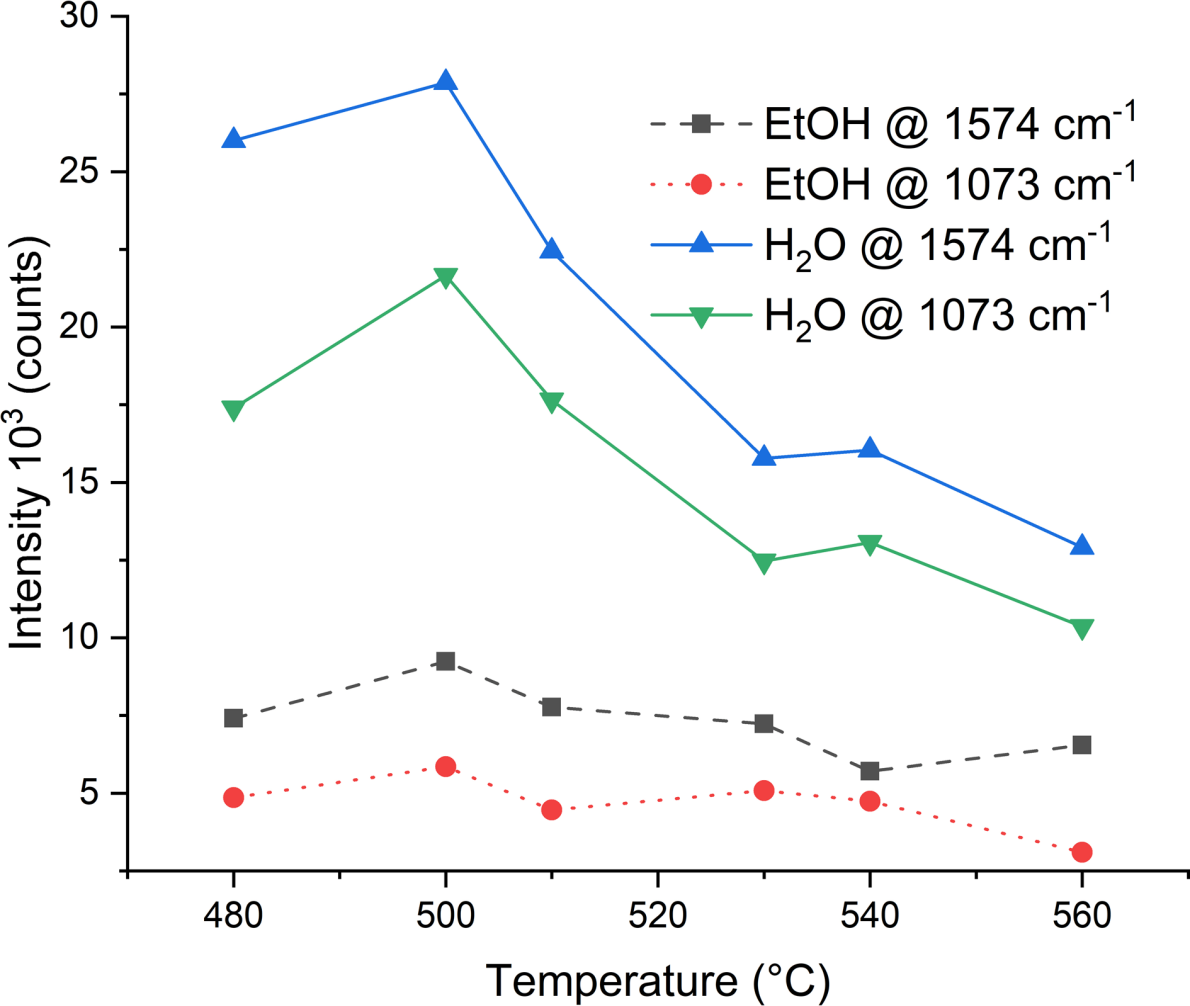
SERS intensities of the 1073 and 1574 cm^−1^ bands before (denoted EtOH) and after H_2_O washing for different VLS process temperatures.

Figure 2 shows the SERS intensities of the 1073 and 1574 cm^−1^ bands before and after H_2_O washing for different VLS process temperatures. The optimal SERS signal is obtained for a temperature of 500 °C during the VLS process, and the SERS signal significantly increases after washing with water.

The first observation can be clearly explained as follows: The temperature during annealing prior to VLS synthesis influences the size and distribution of the Au seeds on the Si wafer, while the VLS process temperature determines growth rate and thickness of the SiNWs [34]. Geometry, density and the changes in surface morphology of the SiNWs influence the variations in SERS intensity.

The second observation can be explained in two ways. Firstly, 4-MPBA interacts with water; and secondly, EtOH or H_2_O capillary forces influence the surface morphology of the substrate. The pH value of 100% ethanol is 7.33, while the water has a pH value equal to 7. Therefore, we do not expect a significant increase in SERS intensity due to the reorientation of 4-MPBA that could be ascribed to a small change of pH value (see the charge transfer and absorbance in [35]). The influence of the capillary forces will be discussed in the following sections.

### Ag decoration and morphology of Si nanowires and immersion-induced changes

**Figure 3:**
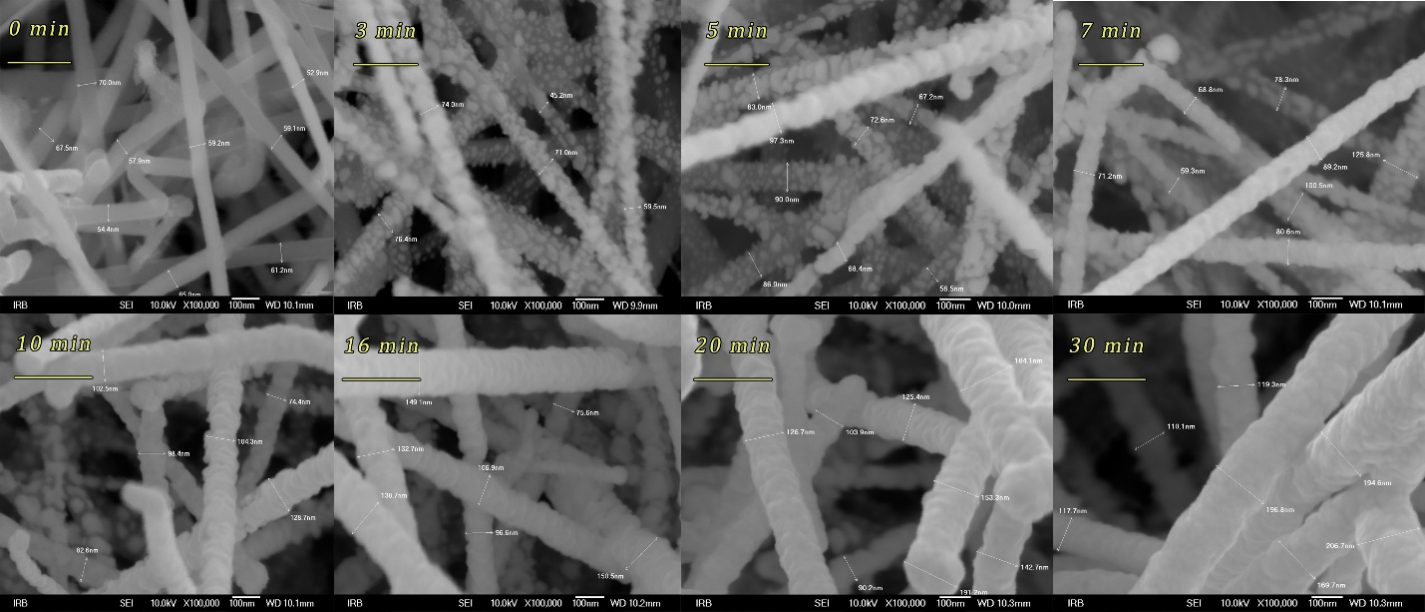
SEM images of horizontally in-plane randomly oriented SiNWs after different Ag sputtering times.

Using 500 °C as the optimal VLS process temperature, we decorated SiNWs through Ag sputtering. The sputtering time varied from 3 to 30 min. The corresponding SEM images recorded at a magnification of 100000× are shown in Figure 3.

The thickness of the SiNWs was measured at several points for each sputtering time and the average values are given in Figure S5 in Supporting Information File 1. It is shown that the thickness linearly increases with the sputtering time and these average values are used equivalently to the sputtering times in the remainder of the paper. The non-sputtered SiNWs have an average thickness of around 60 nm. In Figure 10 of [26], the authors reported approximately the same thickness of 60 nm after 1 h of VLS deposition at 500 °C.

**Figure 4:**
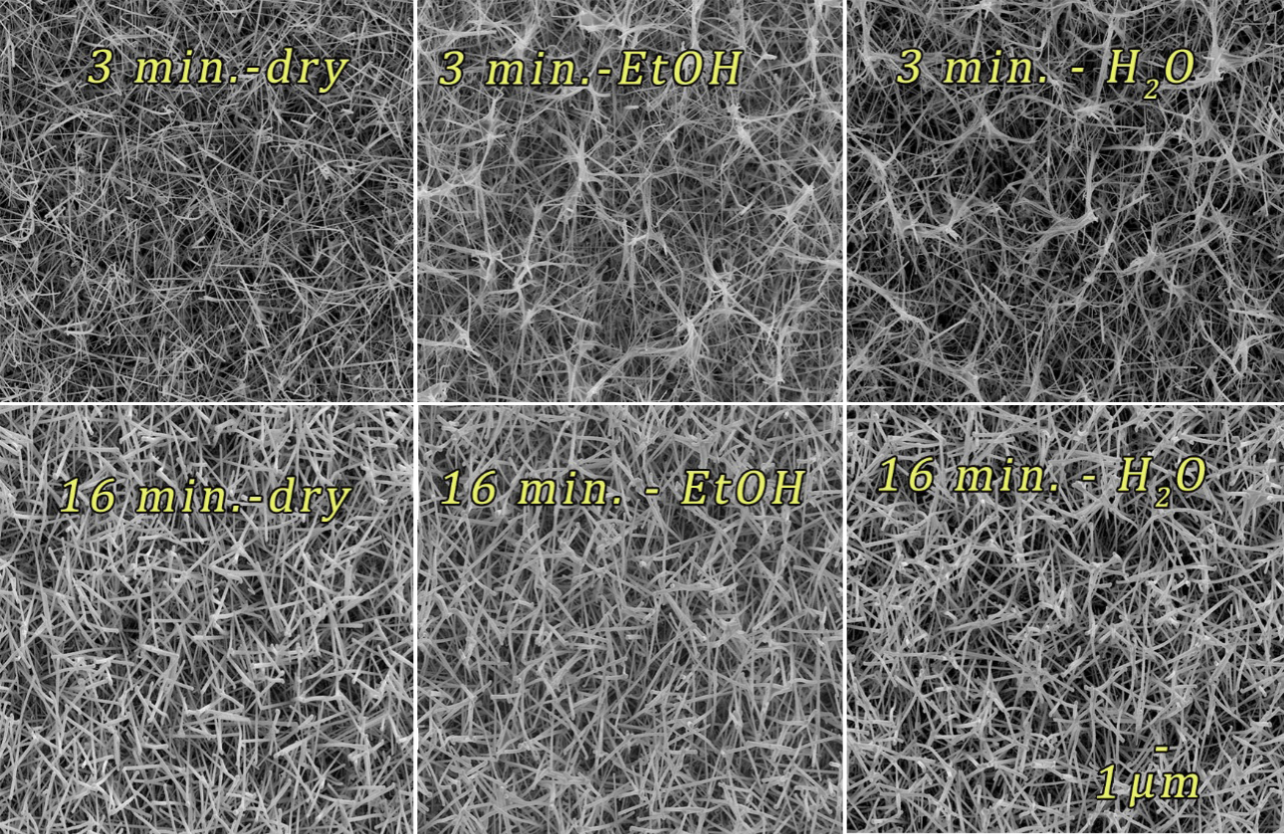
SEM images of dry samples in comparison with the one after immersion in EtOH and water for two different sputtering times of 3 and 16 min.

After short sputtering times (3 and 5 min), the SiNWs are decorated with irregularly shaped droplets of 20–60 nm diameter (Supporting Information File 1, Figure S6). In the range from 7 to 10 min (Figure 3), the upper SiNW layer is completely covered with Ag, yielding Ag cylinders for SERS while in the lower SiNW layers there are only Ag nanoparticles. The lower SiNW layers contribute less to SERS amplification than the upper layer. Sputtering for 16 to 30 min completely covered the SiNWs with Ag, while the thickness increased with the sputtering time. The samples shown in Figure 3 were immersed in an ethanol solution of 4-MPBA and were subsequently washed in water. The complete set of SEM images is presented in Supporting Information File 1, Figures S7–S9, while selected images are given in Figure 4.

**Figure 5:**
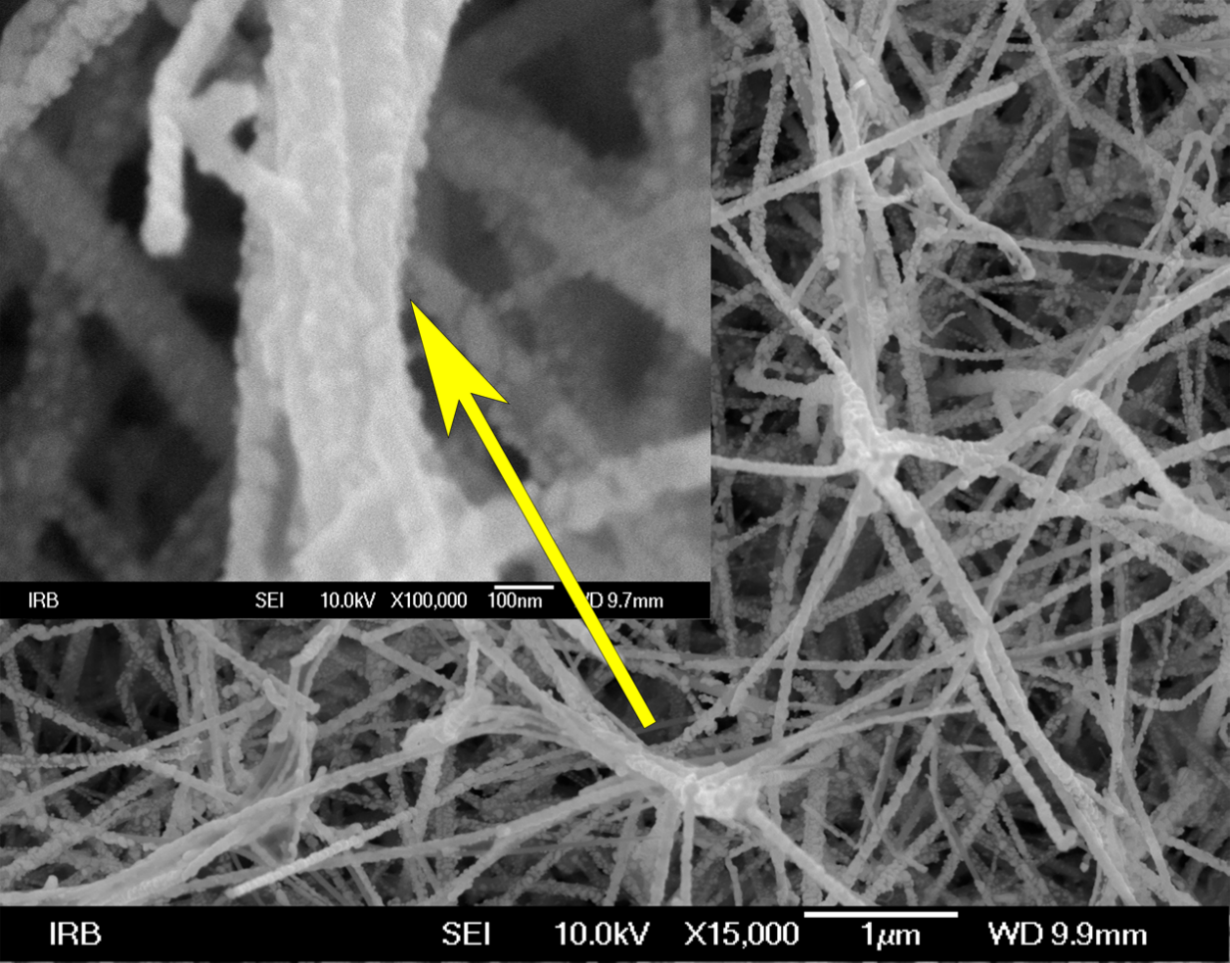
SEM images of SiNWs obtained through VLS deposition at 500 °C, then sputtered Ag for 3 min and finally immersed in H_2_O.

The first row in Figure 4 shows SiNWs sputtered with Ag for a time of 3 min. The first image shows the dry sample while the second and the third image show the same sample after immersion in ethanol and water. From these Figures, it can be concluded that the liquid immersion strongly influences the surface morphology of the SiNWs. Figure S10 in Supporting Information File 1 corroborates this observation. Ethanol and especially water pull the SiNWs together creating irregularly shaped bundles (Figure 5). To support this important observation the SiNWs bundles are shown for different sputtering times in Supporting Information File 1, Figure S11. Furthermore, a shorter deposition time allows the SiNWs to move much easier than longer sputtering times (more than 16 min). From the comparison of the samples sputtered for 16 min, no significant change can be observed regardless the liquid immersion. Therefore, a sputtering time longer than 16 min (Supporting Information File 1, Figures S7–S9 and Figure S11) fixates the SiNWs, not allowing them to form bundles.

Flexibility of the vertical SiNWs can be achieved by a certain aspect ratio of SiNWs. In [36] the leaning of ca. 32 µm long and 80–200 nm thick SiNWs was observed prior to measurements. Flexible SiNWs with a different aspect ratio of 1:10, (100 nm in diameter and 1.0–1.3 µm in height) were reported in [37]. “Leaning fingertips” features were claimed in commercially available substrates [20] where the aspect ratio was ca. 1:15 with significantly shorter and thinner SiNWs (600 nm height and 40 nm thickness). Drawing the parallel between the horizontal and vertical SiNWs, we have also observed that the SiNW thickness influences the flexibility, i.e., the ability of SiNWs to bundle together. An even more important factor was Ag plating, which freezes the SiNWs contact points not allowing them to move. The length of horizontal SiNWs does not play a crucial role regarding the flexibility, but it is very important in order to give a high SiNWs surface density, which consequently guarantees uniform SERS signals at different locations of the substrate.

A closer look (Supporting Information File 1, Figure S10) shows that water has a stronger impact than ethanol on the surface morphology. This can be explained by the higher average number of hydrogen bonds in water (ca. 3.8) than in ethanol (ca. 2) and the, consequently, stronger surface tension, 72.86 and 22.39 mN·m^−1^ at 20 °C, respectively [38–39]. SiNWs are captured by the water surface tension through adhesive forces [40] and during drying, the strong surface tension moves the SiNWs towards each other creating twisted and irregular SiNWs bundles. For the long sputtering times (when the SiNWs are completely covered with Ag), adhesion and surface tension are not strong enough to overcome the stiffening caused by sputtering.

Another significant substrate feature is surface wetting. Unlike vertical SiNWs [38], horizontal SiNWs are hydrophilic, as freshly prepared SiNW substrate as well as after Ag sputtering (Supporting Information File 1, Figure S12). The reasons are predominantly the characteristic surface roughness and the chemical affinity. Generally, the hydrophilic substrate surface is desirable for hydrophilic molecules such as for example dextrose and albumin [41].

### SERS sensing of 4-MPBA

In order to determine the optimal Ag-sputtering time, we measured SERS spectra for four different 4-MPBA concentrations (10^−3^, 10^−4^, 10^−5^ and 10^−6^ M) at 100 different points. The mapping points, separated by 10 µm, were spaced in a 100 × 100 µm grid. Also, all samples were measured before and after immersion in H_2_O.

**Figure 6:**
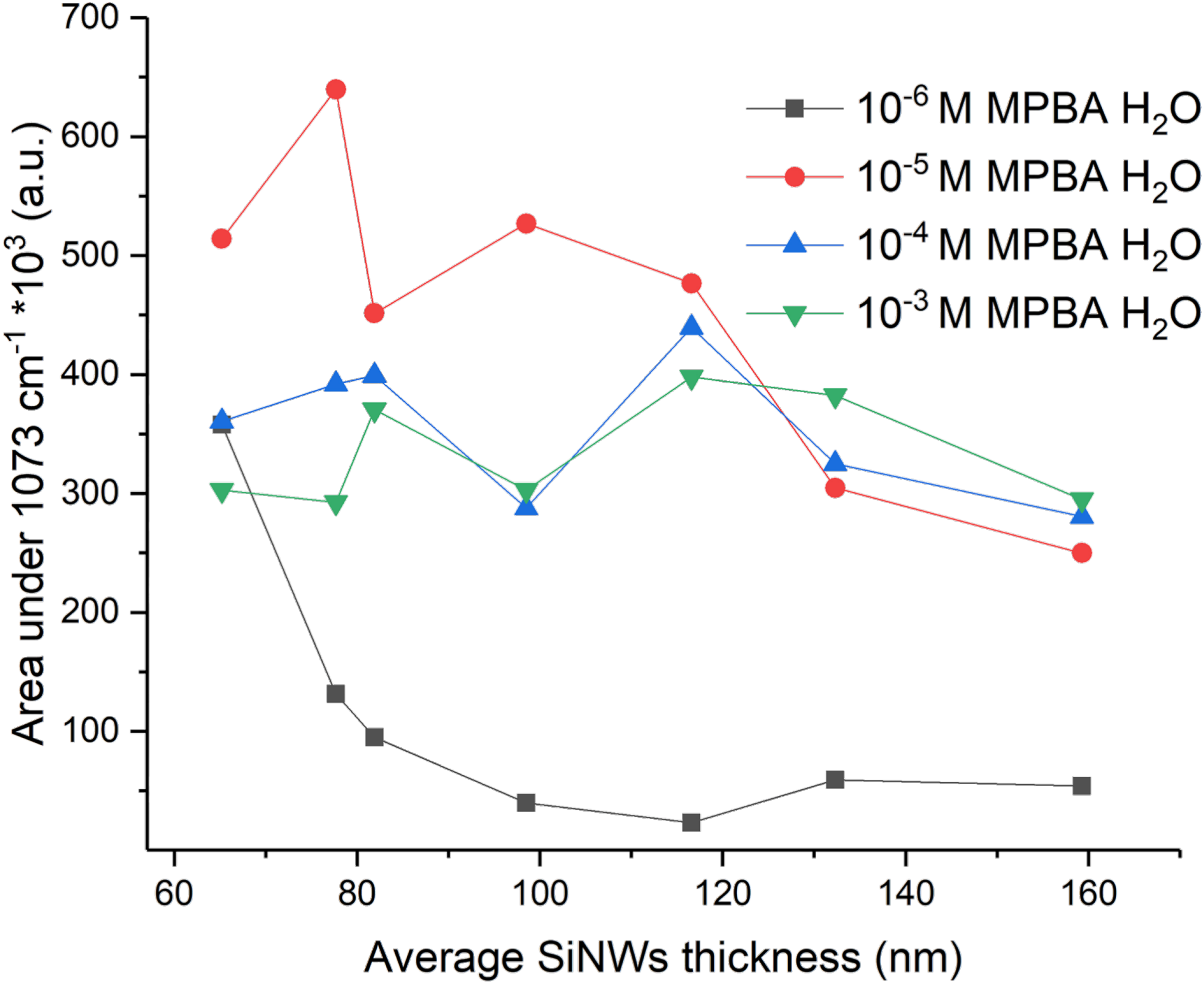
Average SERS values of 4-MPBA for different SiNW thicknesses after H_2_O immersion.

The average SERS values of 4-MPBA ethanol solutions at different SiNWs thicknesses are shown in Supporting Information File 1, Figure S13. Figure 6 shows the average SERS values only after immersion in H_2_O. The standard deviation was significantly higher for the lower (10^−6^ M) than for the higher (10^−5^ to 10^−3^ M) concentrations of 4-MPBA (Supporting Information File 1, Figure S14). The sample homogeneity is also shown by a colored-pixel map (Supporting Information File 1, Figure S14).

The SERS intensity not only depends on the SiNW thickness (sputtering time), but also on the 4-MPBA concentration. Adding more analyte will not increase the SERS intensity as much as it did between 10^−6^ and 10^−5^ M. It indicates that there is only a certain number of possible active sites on Ag that can host the analyte molecules. The reorientation of 4-MPBA or a shielding of the first analyte monolayer could also contribute to this effect.

At a concentration of 10^−6^ M, the best SERS results are achieved for thin SiNWs, while for higher concentrations the SERS intensity is quite constant up to ca. 120 nm thickness, after which it starts decreasing. The intensity decrease with increasing thickness shows that not only metal nanoparticle size and SiNW thickness are important for SERS enhancement, but also the quality of the hot spots. We can see that when Ag sputtering freezes the SiNW structure, SiNWs cannot aggregate to bundles and consequently the SERS intensity decreases. A possible shift of the localized surface plasmon absorption band is out of the scope of this paper.

**Figure 7:**
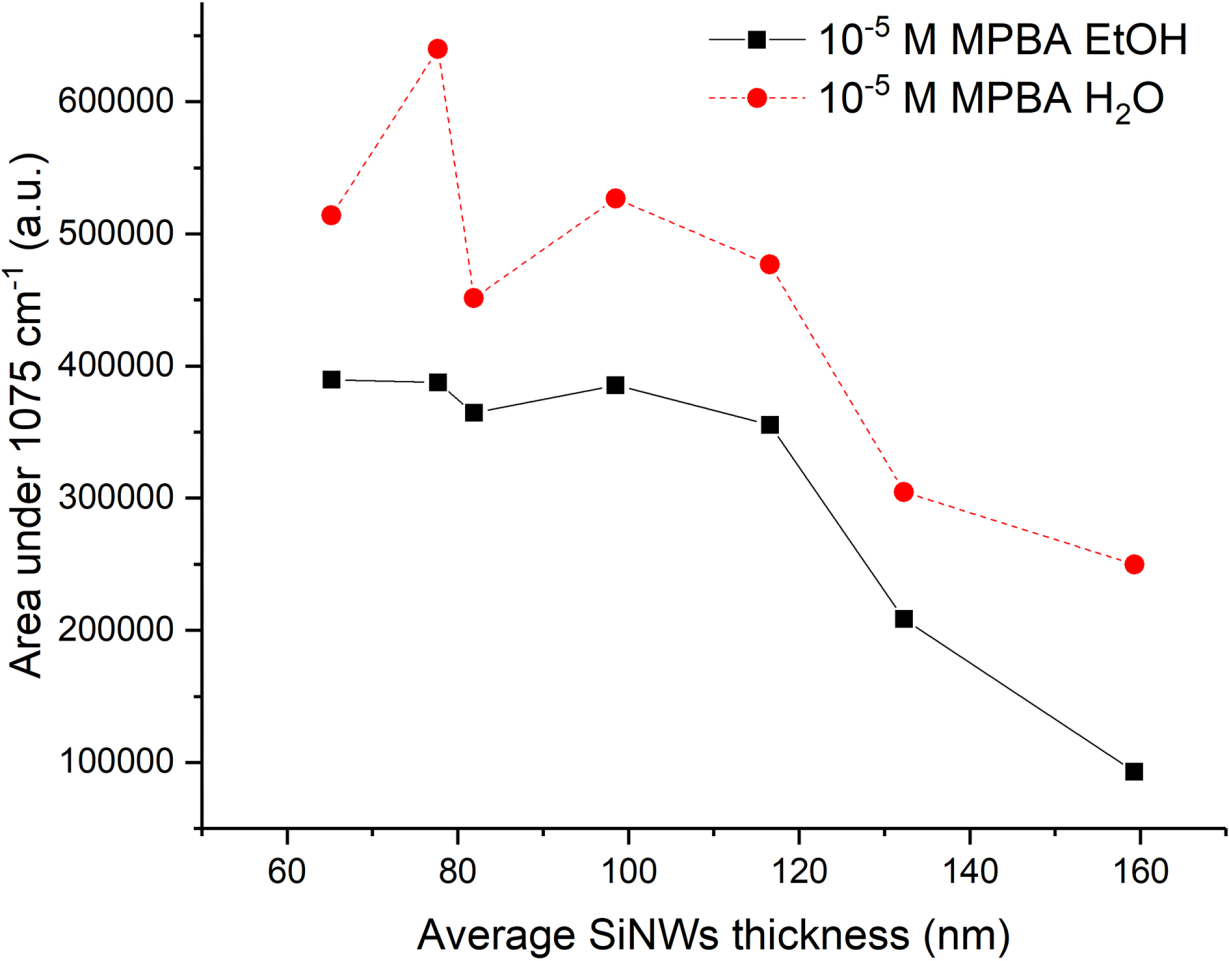
SERS enhancement after immersion in water.

We can see a significant increase of the SERS signal at 10^−5^ M after water immersion (Figure 7). The same behavior is observed for other concentrations, however, sometimes the difference between the SERS intensities before and after water immersion is small (Supporting Information File 1, Figure S16). As reported in [42] we assume that capillary forces dominate over van der Waals forces by several orders of magnitude. During drying, the adhesion between liquid and SiNW surface pulls and bends the SiNWs, changing the substrate morphology and consequently increasing the SERS intensity. Water has a higher surface tension than ethanol and, consequently, pulls the SiNWs together stronger causing a larger SERS enhancement. Since the SERS effect decreases with distance [43], bringing the SiNWs closer significantly improves the analyte detection. In [2] the author assumed that the enhancement factor increases approximately as *d*^−8^ in the case of two metal nanoparticles with the polarization along the particle axis, which can be roughly applied to the case of two nanowires. However, in the reported substrates SiNWs are randomly oriented and the polarization measurement was not tested in detail.

### Fractal dimension and lacunarity of Ag-plated SiNWs

#### Fractal dimension

**Figure 8:**
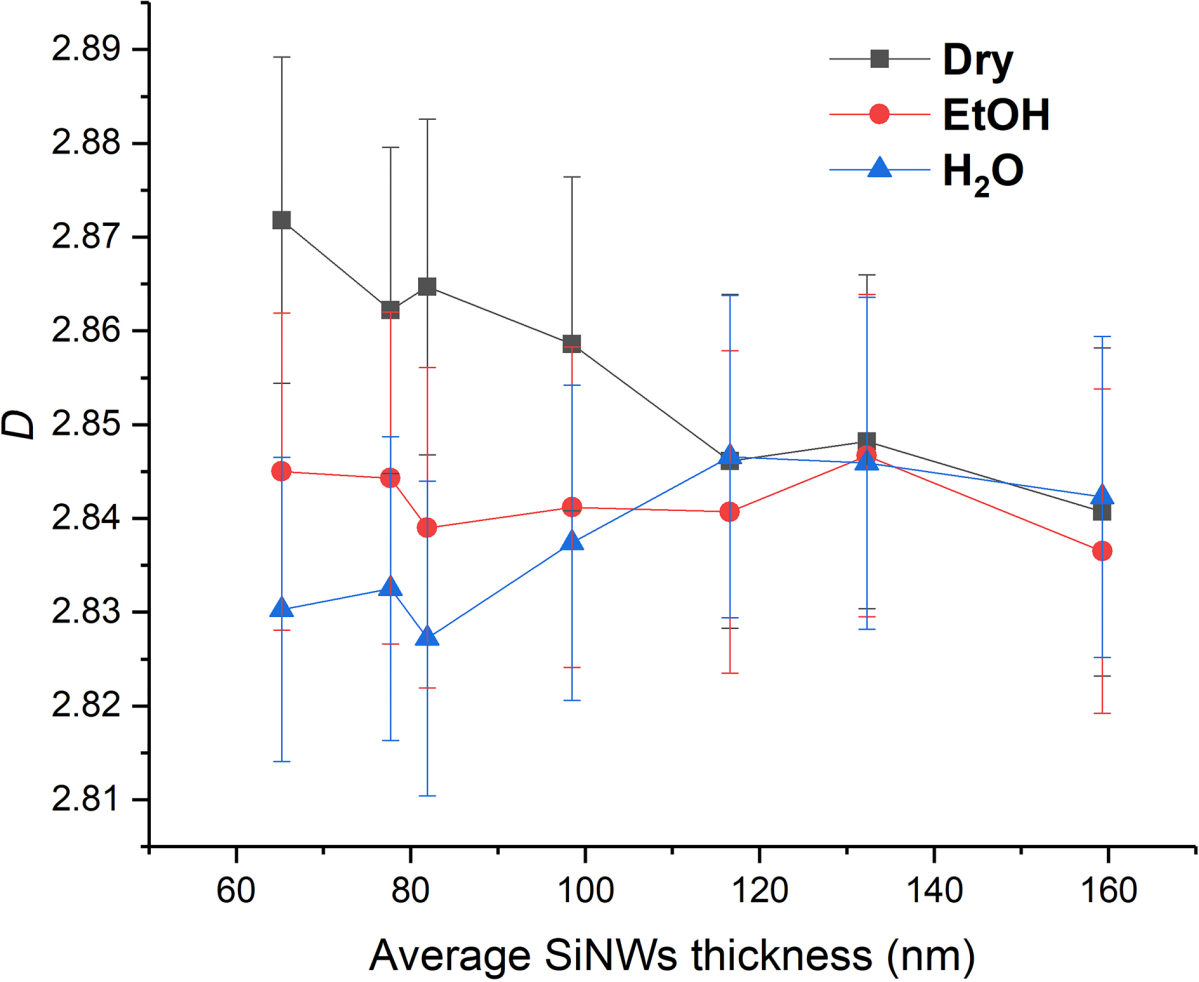
Fractal dimension (*D*) of Ag-plated SiNWs after immersion in EtOH and H_2_O.

In order to describe horizontal SiNW morphology in more detail, we calculated the average fractal dimension (*D*) from the SEM images (Supporting Information File 1, Figures S7–S9) for all SERS-active samples (Figure 8). FracLac delivers a measure of the box-counting fractal dimension, which is the measure of complexity, i.e., the change in detail with a change in scale. The average value of *D* is the usual box-counting fractal dimension averaged over the number of scans that carried out done at different grid positions [25]. For three-dimensional objects, the expected *D* values are between 2 and 3.

Figure 8 clearly shows that the fractal dimension decreases after the EtOH and H_2_O immersion (*D*_dry_ > *D*_EtOH_ > *D*_water_) for samples with an average SiNWs thickness below 120 nm. For average SiNWs thickness values of 120–160 nm, there is no change of the fractal dimension. Correlating these results with the SEM images (Supporting Information File 1, Figures S7–S9), one can see that Ag sputtering freezes the SiNWs structure and the fractal dimension remains constant. The second conclusion is that as the water pulls the SiNWs together (Supporting Information, File 1, Figure S10), the fractal dimension decreases (Figure 8). This leads to the creation of hot spots along the SiNWs which result in an enhanced SERS effect (Figure 7). After immersion in a liquid, there is not only a SiNW redistribution in the *xy*-plane, but also along the *z*-axis. However, this aspect is beyond the scope of this paper.

#### Lacunarity

**Figure 9:**
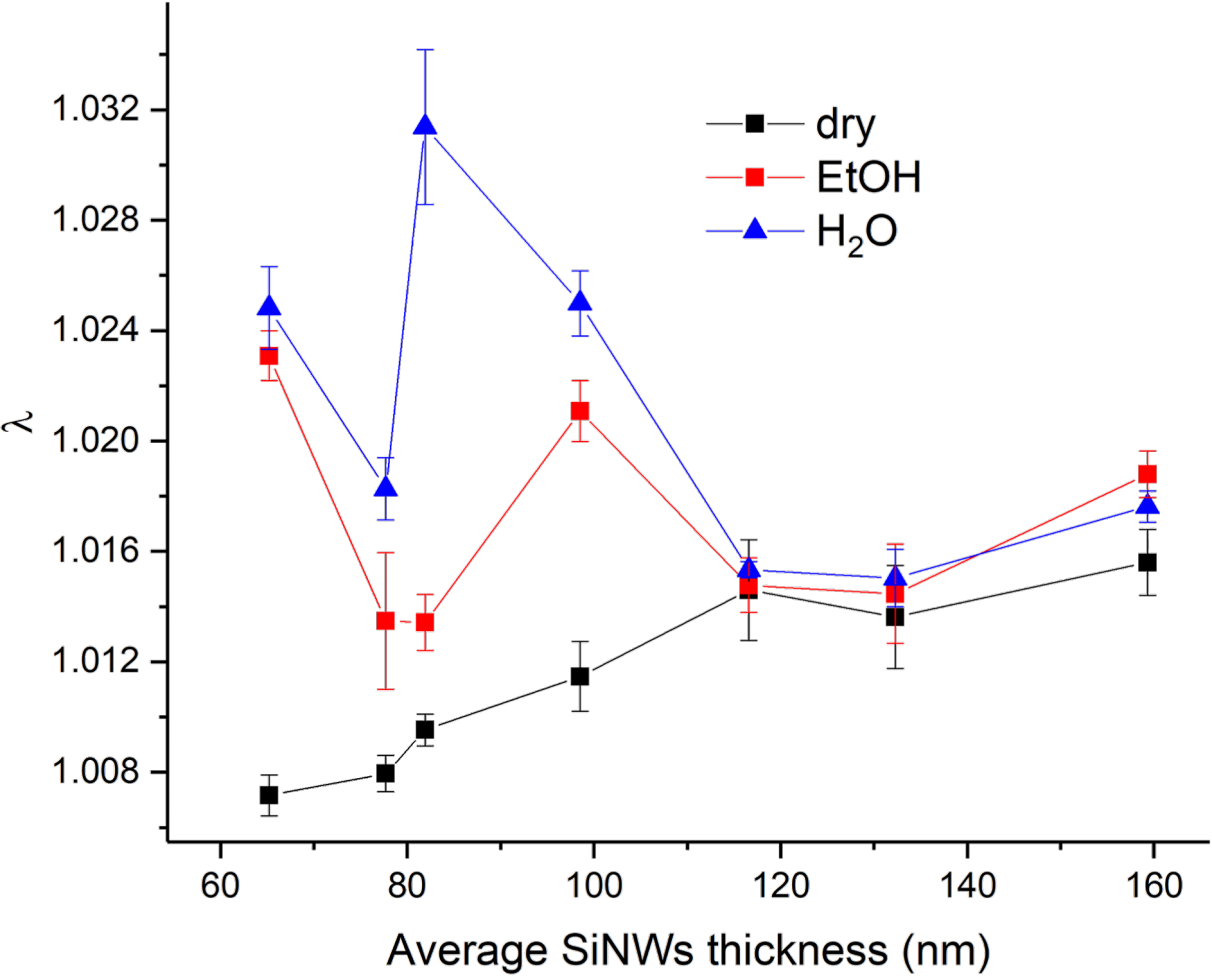
Lacunarity at 532 nm for different SiNWs thicknesses.

Complementary to fractal dimension, lacunarity gives additional morphological information. As the cross junctions between AgNPs and SiNWs are important for the creation of hot spots and SERS enhancement, the nanosized gaps between SiNWs can behave as a resonant cave where the incident laser light is scattered numerous times further contributing to SERS amplification. Those voids can be described by lacunarity, which is considered a measure of heterogeneity (inhomogeneity) or translational or rotational invariance in an image [25]. Lacunarity values at the excitation wavelength of 532 nm are calculated as described in Supporting Information File 1 and shown in Figure 9.

The lacunarity of dry substrates increases after immersion in EtOH and water. The lacunarity confirms the assumption drawn from the SEM images that the long sputtering time freezes the SiNW structure, making it impervious to immersion in liquid. Similar lacunar values for the three thickest samples are observed (Figure 9), analogous to the calculations of fractal dimension (Figure 8). Furthermore, the fractal dimension decreases after the immersions for thinner samples while the lacunarity increases. The decrease of the fractal dimension is the consequence of the SiNWs flexibility and their tendency to bundle together. Consequently, it leaves bigger gaps that do not have the same width of the size distribution as in the case of dry samples. There are wider and different gap sizes resulting in the lacunarity increase. The analysis of fractal dimension and lacunarity shows the results after water immersion are more separated from the dry sample than the results after ethanol immersion, indicating that water places the SiNWs closer together than EtOH. The smaller the gaps between the SiNWs result in a stronger SERS effect.

#### Comparison with commercially available SERS substrates

**Figure 10:**
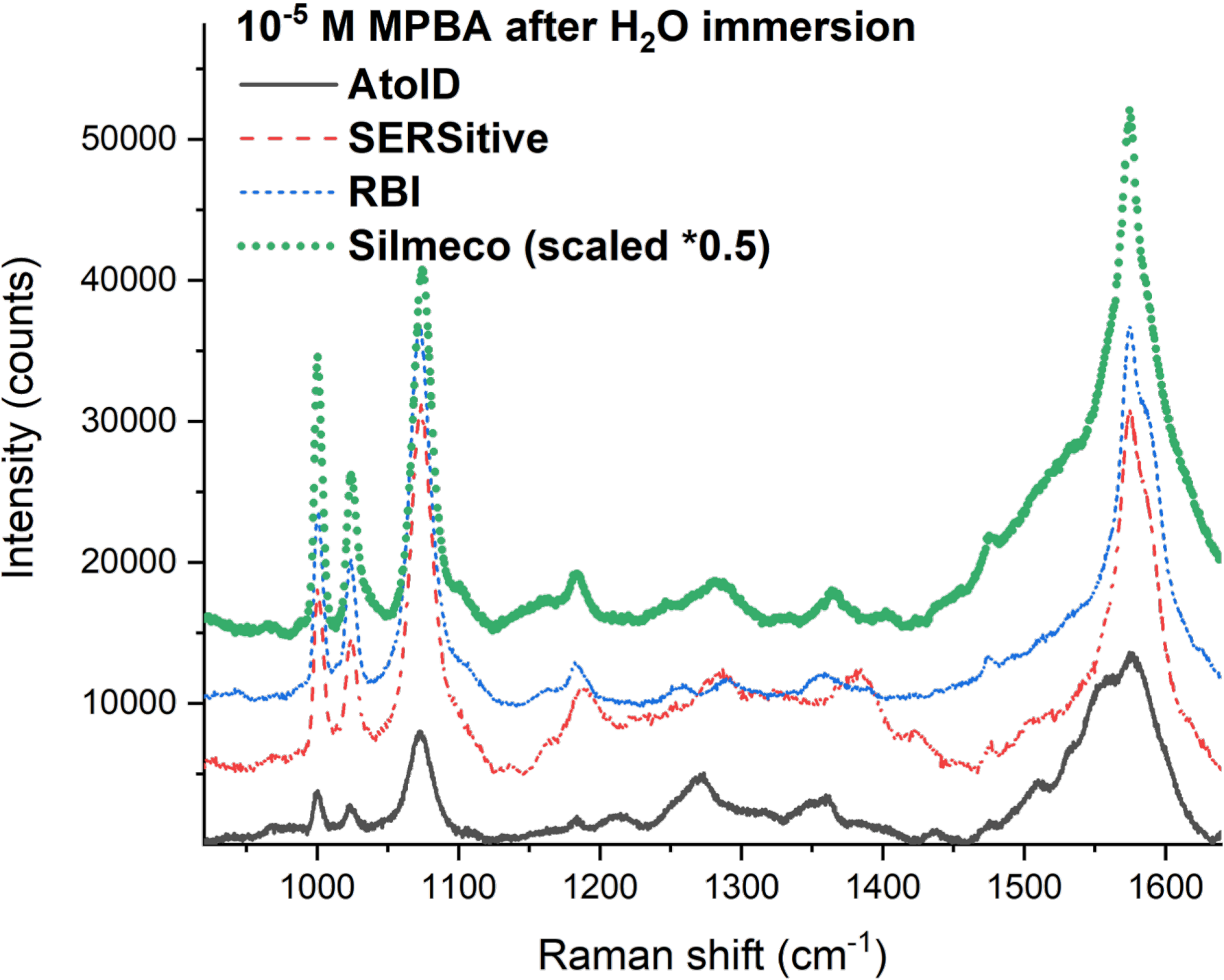
Comparison of the commercial SERS substrates and the synthetized sample (RBI) after the immersion in water.

The synthesized samples were compared with commercially available samples utilizing 10^−5^ M 4-MPBA solution in ethanol as analyte. SERS measurements were carried out on the same samples two times. The first one immediately after drying of EtOH (Supporting Information File 1, Figure S18) and the second one after immersion in milli-Q water and subsequent drying (Figure 10). The first SERS measurement showed similar values for all three commercially fabricated samples with somewhat lower values of our RBI lab sample.

After immersion in water, the SERS intensity was drastically different. Of all three samples used for the comparison only Silmeco has flexible SiNWs. Their SERS values showed a 2–3-times stronger intensity in comparison to the RBI spectrum recorded under the same conditions. Our lab sample showed a significant increase after immersion in water as well and becomes more than comparable with AtoID and SERSitive substrate spectra. A more detailed comparison between Silmeco and our lab samples is given in Supporting Information File 1, Figure S19.

**Figure 11:**
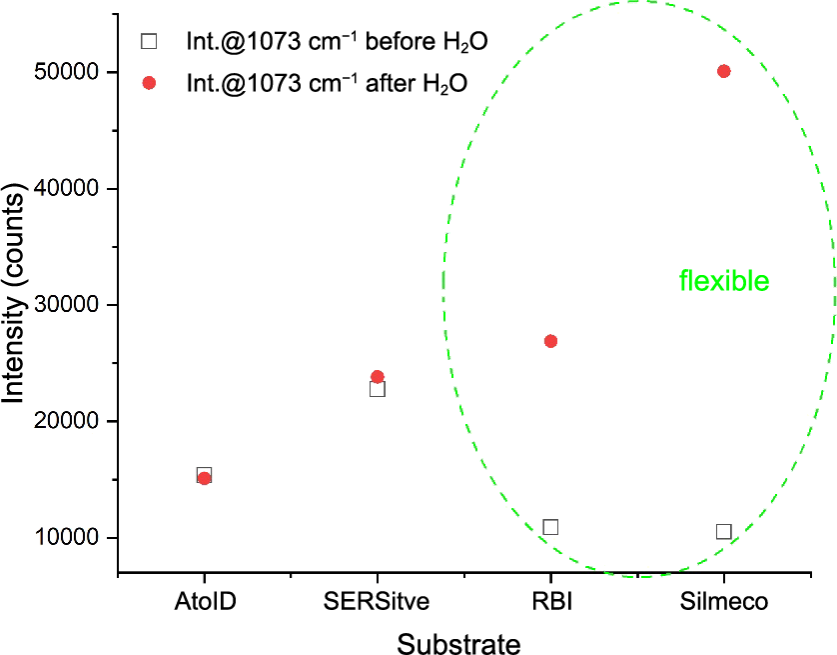
Intensity at 1073 cm^−1^ for the samples fabricated in different labs.

These results show the advantage of the flexible nanostructures and self-assembled hot spots for SERS applications. We note here that the comparison of the commercially available SERS substrates is obtained on a reduced number of samples without detailed mapping and therefore is not a subject of the SERS market rating (Figure 11).

## Conclusion

This research showed the advantages of SERS substrates with flexible silicon nanowires over solid substrates with a fixed structure. The fabrication process and impact of each preparation stage are presented in detail. It is shown that the optimal SiNWs thickness decorated with Ag is in the range from 60 to 100 nm. This thickness allows for the flexibility of the several micrometers long, horizontally placed and randomly oriented SiNWs. The strong SERS enhancement mechanism relies on bringing the SiNWs to nanogap-vicinity, which creates a system comparable to optical tweezers and allows for localized surface plasmons and strong electric fields to occur. The morphological surface changes after immersion in ethanol in water are described by analyzing scanning electron images, particularly by using fractal and lacunar analysis. The corresponding fractal dimensions and lacunarity at excitation wavelength are both not only compliant with each other, but also with SERS measurements. This result strongly encourages researchers to describe the solid substrates with fractal and lacunar information since they could correlate morphology and SERS measurements results. To the best of our knowledge, we have not seen detailed reports on SERS substrates with horizontally placed flexible silicon nanowires. The comparison with commercially available substrates utilizing 4-MPBA as a test molecule showed that these samples keep pace with the best SERS market products. These preliminary results are promising and encourage for further improvements.

## Supporting Information

File 1 Additional experimental data.

## References

[1] Fleischmann M, Hendra P J, McQuillan A J (1974). Chem Phys Lett.

[2] Moskovits M (2005). J Raman Spectrosc.

[3] Sujith A, Itoh T, Abe H, Yoshida K-i, Kiran M S, Biju V, Ishikawa M (2009). Anal Bioanal Chem.

[4] Blackie E J, Le Ru E C, Etchegoin P G (2009). J Am Chem Soc.

[5] Zrimsek A B, Wong N L, Van Duyne R P (2016). J Phys Chem C.

[6] Lane L A, Qian X, Nie S (2015). Chem Rev.

[7] Wang Y, Irudayaraj J (2013). Philos Trans R Soc, B.

[8] Rae S I, Khan I (2010). Analyst.

[9] Schlücker S (2014). Angew Chem, Int Ed.

[10] Pang S, Yang T, He L (2016). TrAC, Trends Anal Chem.

[11] Janči T, Mikac L, Ivanda M, Marušić Radovčić N, Medić H, Vidaček S (2017). J Raman Spectrosc.

[12] Xie X, Pu H, Sun D-W (2018). Crit Rev Food Sci Nutr.

[13] Kumar S, Goel P, Singh J P (2017). Sens Actuators, B.

[14] Xiu X, Guo Y, Li C, Li Z, Li D, Zang C, Jiang S, Liu A, Man B, Zhang C (2018). Opt Mater Express.

[15] Park S, Lee J, Ko H (2017). ACS Appl Mater Interfaces.

[16] Yang L, Hu J, Bai K (2016). J Adhes Sci Technol.

[17] Wang G, Yi R, Zhai X, Bian R, Gao Y, Cai D, Liu J, Huang X, Lu G, Li H (2018). Nanoscale.

[18] Lu G, Lia H, Zhang H (2011). Chem Commun.

[19] Schmidt M S, Hübner J, Boisen A (2012). Adv Mater (Weinheim, Ger).

[20] Wu K, Rindzevicius T, Schmidt M S, Mogensen K B, Xiao S, Boisen A (2015). Opt Express.

[21] Wong C L, Dinish U S, Schmidt M S, Olivo M (2014). Anal Chim Acta.

[22] Galopin E, Barbillat J, Coffinier Y, Szunerits S, Patriarche G, Boukherroub R (2009). ACS Appl Mater Interfaces.

[23] Gebavi H, Ristić D, Baran N, Mikac L, Mohaček-Grošev V, Gotić M, Šikić M, Ivanda M (2018). Mater Res Express.

[24] Kern W (1990). J Electrochem Soc.

[25] (2018). ImageJ.

[26] Gadea G, Morata A, Santos J D, Dávila D, Calaza C, Salleras M, Fonseca L, Tarancón A (2015). Nanotechnology.

[27] Mosier-Boss P (2017). Nanomaterials.

[28] Schomacker K T, Delaney J K, Champion P M (1986). J Chem Phys.

[29] Wang P, Pang S, Pearson B, Chujo Y, McLandsborough L, Fan M, He L (2017). Anal Bioanal Chem.

[30] Pham X-H, Shim S, Kim T-H, Hahm E, Kim H-M, Rho W-Y, Jeong D H, Lee Y-S, Jun B-H (2017). BioChip J.

[31] Sun F, Bai T, Zhang L, Ella-Menye J-R, Liu S, Nowinski A K, Jiang S, Yu Q (2014). Anal Chem (Washington, DC, U S).

[32] Sun X, Stagon S, Huang H, Chen J, Lei Y (2014). RSC Adv.

[33] Su H, Wang Y, Yu Z, Liu Y, Zhang X, Wang X, Sui H, Sun C, Zhao B (2017). Spectrochim Acta, Part A.

[34] Gebavi H, Ristić D, Baran N, Mikac L, Mohaček-Grošev V, Gotić M, Ivanda M (2018). Silicon.

[35] Su H, Wang Y, Yu Z, Liu Y, Zhang X, Wang X, Sui H, Sun C, Zhao B (2017). Spectrochim Acta, Part A.

[36] Zhang M-L, Fan X, Zhou H-W, Shao M-W, Zapien J A, Wong N-B, Lee S-T (2010). J Phys Chem C.

[37] Kara S A, Keffous A, Giovannozzi A M, Rossi A M, Cara E, D'Ortenzi L, Sparnacci K, Boarino L, Gabouze N, Soukane S (2016). RSC Adv.

[38] Pallas N R, Harrison Y (1990). Colloids Surf.

[39] Adamson A W, Gast A P (1997). Physical chemistry of surfaces.

[40] Bowen J, Rossetto H L, Kendall K (2016). Surf Topogr: Metrol Prop.

[41] Pérez-Mayen L, Oliva J, De la Rosa Cruz E (2014). Selection criteria for SERS substrates. Latin America Optics and Photonics Conference.

[42] DelRio F W, de Boer M P, Phinney L M, Bourdon C J, Dunn M L Van der Waals and Capillary Adhesion of Microelectromechanical Systems. ASME 2006 International Mechanical Engineering Congress and Exposition.

[43] Kovacs G J, Loutfy R O, Vincett P S, Jennings C, Aroca R (1986). Langmuir.

