# Supplementary material for: Features and advantages of flexible silicon nanowires for SERS applications
Source: Beilstein J Nanotechnol. 2019 Mar 15;10:725–34. doi: 10.3762/bjnano.10.72 (PMC6423564; doi:10.3762/bjnano.10.72)
Supplement: File 1 — Additional experimental data. [file Beilstein_J_Nanotechnol-10-725-s001.pdf]

BEILSTEIN JOURNAL OF NANOTECHNOLOGY

Supporting Information

for

Features and advantages of flexible silicon nanowires for SERS applications

Hrvoje Gebavi, Vlatko Gašparić, Dubravko Risović, Nikola Baran, Paweł Henryk Albrycht and Mile Ivanda

Beilstein J. Nanotechnol. **2019,** *10,* 725–734. doi:10.3762/bjnano.10.72

Additional experimental data

License and Terms: This is a supporting information file under the terms of the Creative Commons Attribution License (http://creativecommons.org/licenses/by/4.0). Please note that the reuse, redistribution and reproduction in particular requires that the authors and source are credited.
The license is subject to the *Beilstein Journal of Nanotechnology* terms and conditions: (https://www.beilstein-journals.org/bjnano)

Dependence of SERS intensity on the VLS process temperature

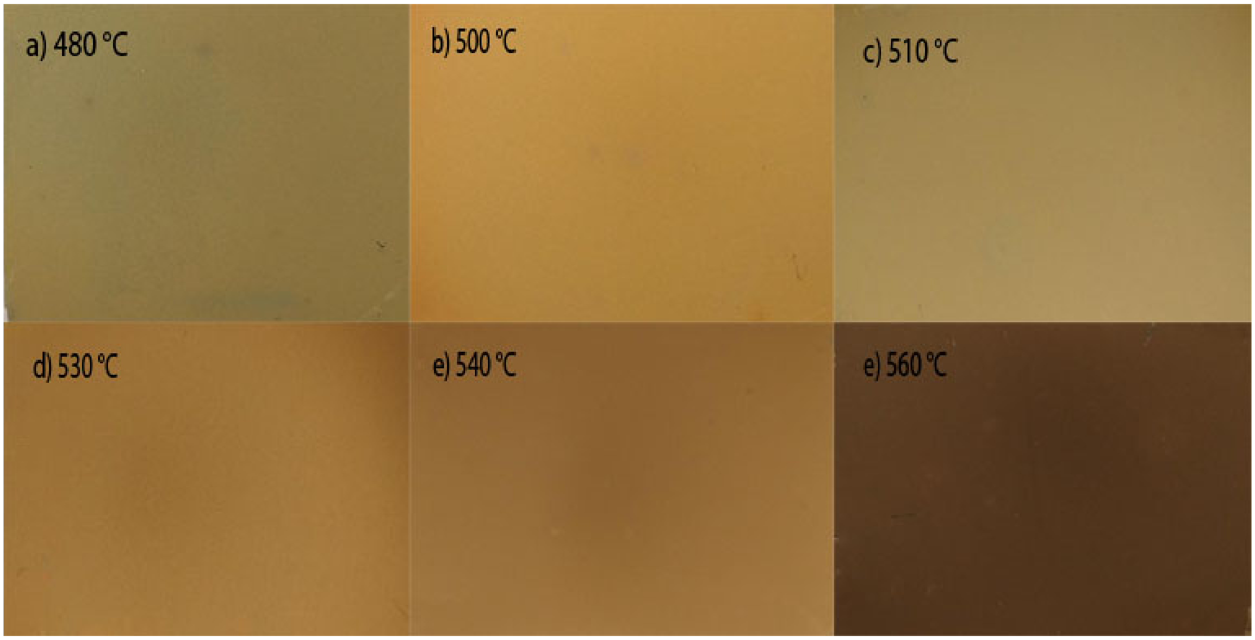

Figure S1: SiNWs color change with VLS temperature.

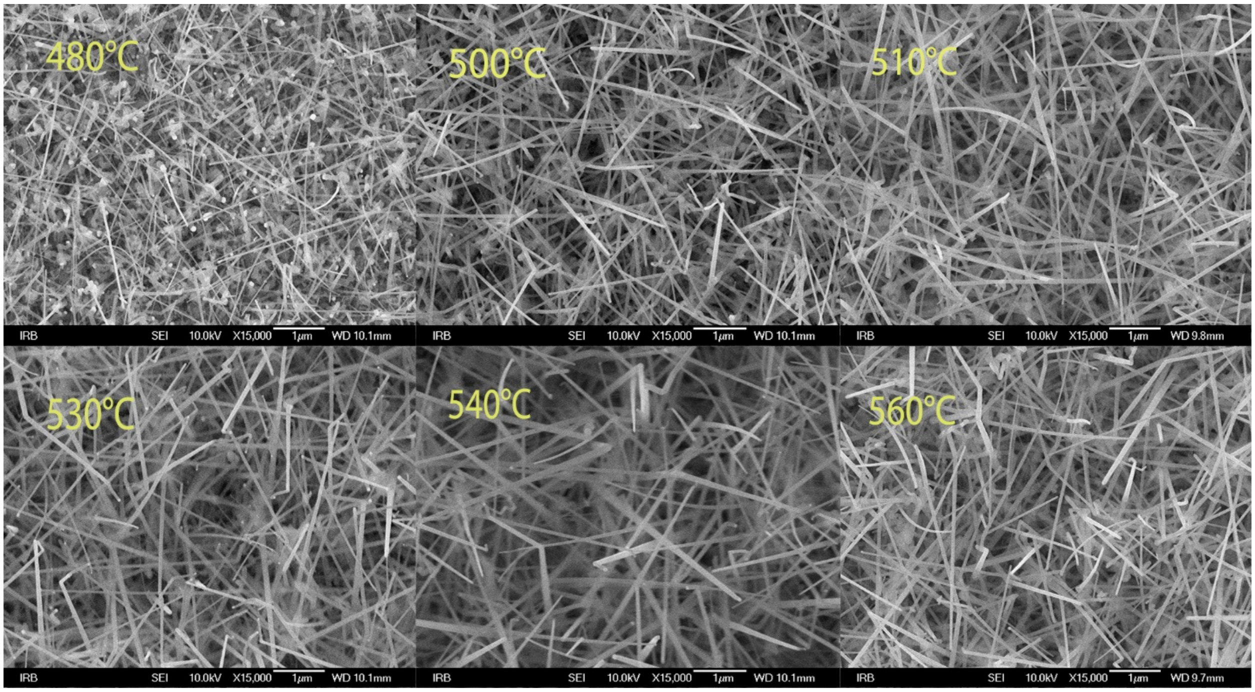

Figure S2: SEM images of SiNWs obtained for various VLS temperatures (corresponding to Figure S1).

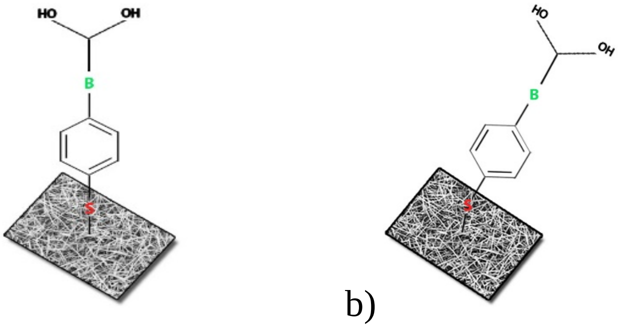

a) b)

Figure S3: a) 4-MPBA molecule attached to the Ag-decorated SiNWs through the thiol group. b) 4-MPBA tilts at acidic pH values.

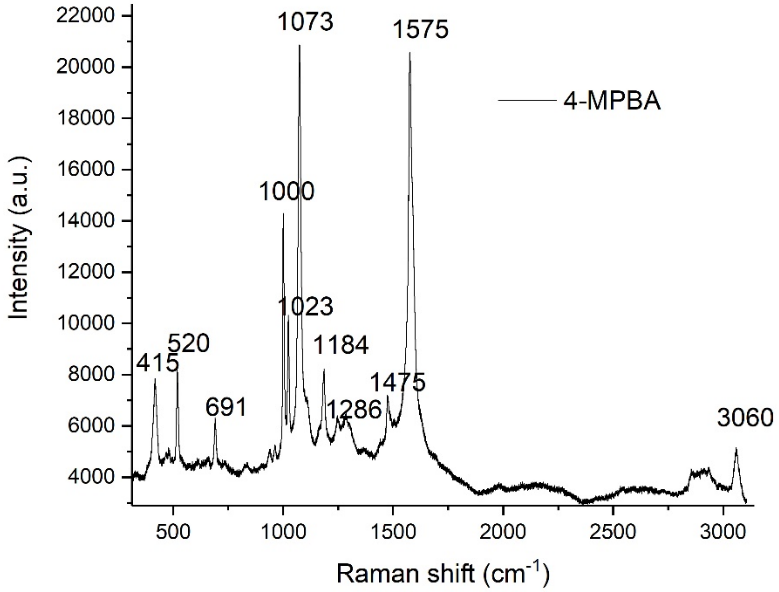

Figure S4: SERS spectrum of 10^{-4} M 4-MPBA.

Table S1: 4-MPBA molecule band assigments.

Observed peak position	Tentative Assignment	Reference
415 cm^{-1}	7a + ν_{CS}	[1]
520 cm^{-1}	Si phonon	[2]
691 cm^{-1}	$\beta_{CH}+\nu_{CS}$<br><br>-in plane bending of the C–C ring and C–S stretching	[3]<br><br>[4]
1000 cm^{-1}	-β_{CCC}<br>-B–O–H deformation vibration<br>-in plane bending of the C–C ring	[3]<br>[5]<br><br>[4]
1023 cm^{-1}	β_{CH} in plane bending	[3]
1073 cm^{-1}	ring C–C bending and C–S stretching	[4,6]
1184 cm^{-1}	ν_9 (a_1)	[7]
1286 cm^{-1}	in-plane bending vibration of B-OH	[8]
1475 cm^{-1}	C–C stretching	[4]
1575 cm^{-1}	ring C–C stretching nontotally symmetric 8b mode	[3-6]
3060 cm^{-1}	C–H stretching[a]	[9]

[a]A weak C–H stretching band at 3060 cm^{-1} might be associated with a flat orientation of the benzoic ring while a more intense band hints at different orientations [9].

Ag decoration and morphology of Si nanowires and immersion-induced changes

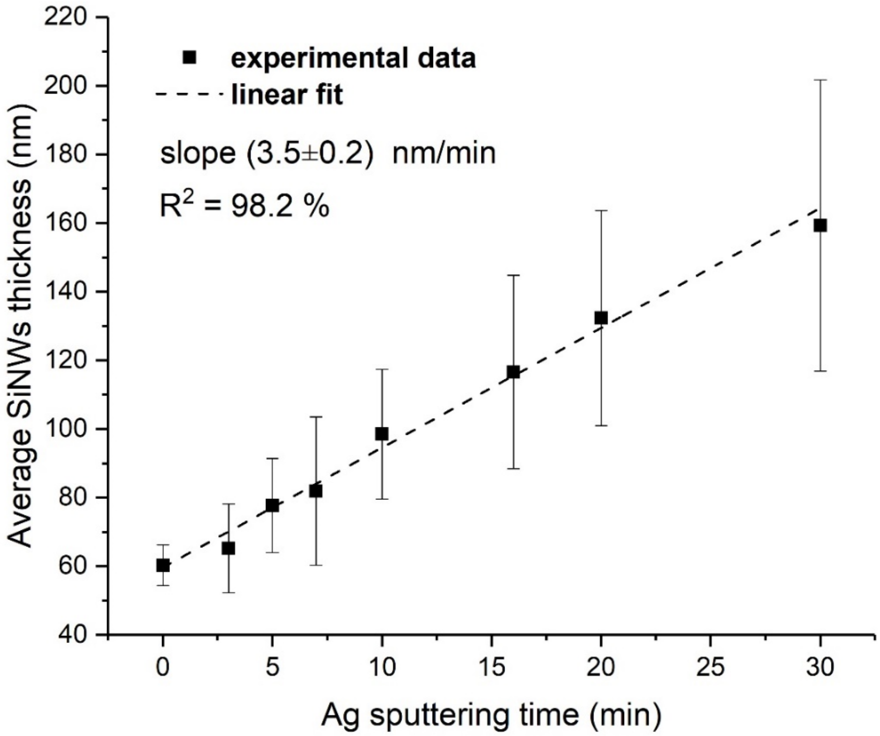

Figure S5: Average SiNW thickness values as a function of the sputtering time.

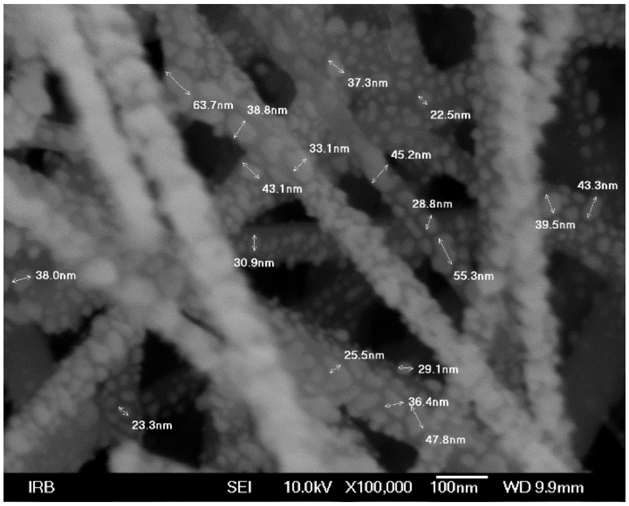

Figure S6: SEM image after 3 min of Ag sputtering. Grain sizes: 20–60 nm.

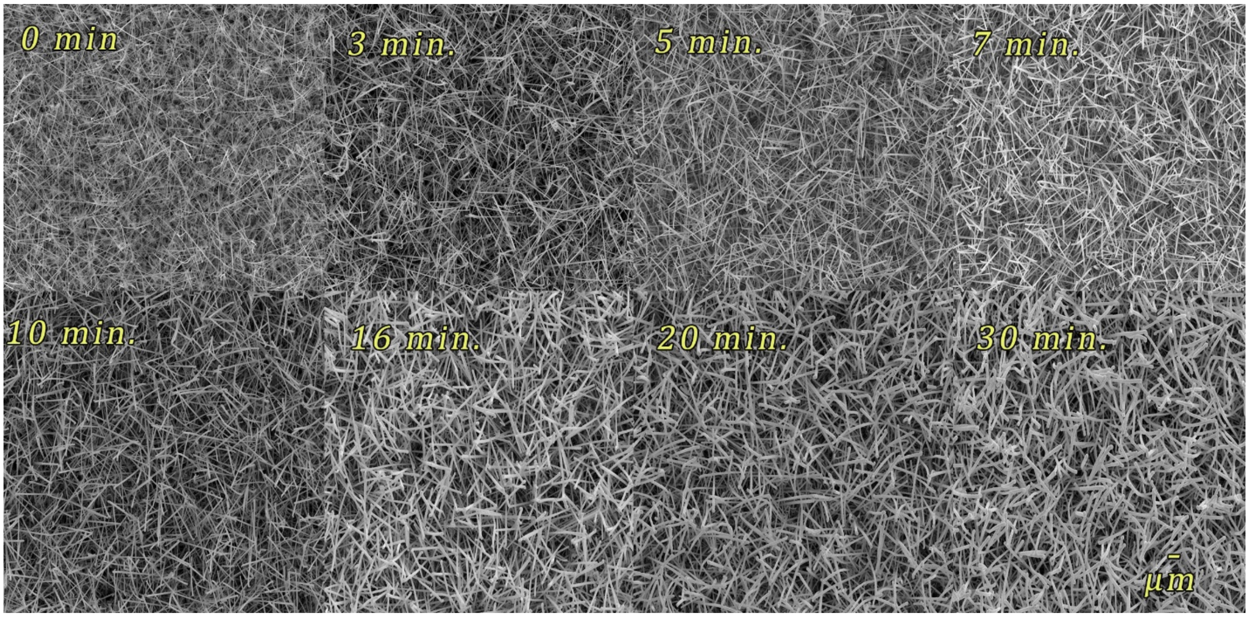

Figure S7: SEM images of dry samples: SiNWs obtained at 500 °C by VLS deposition. Ag sputtering time: 3–30 min.

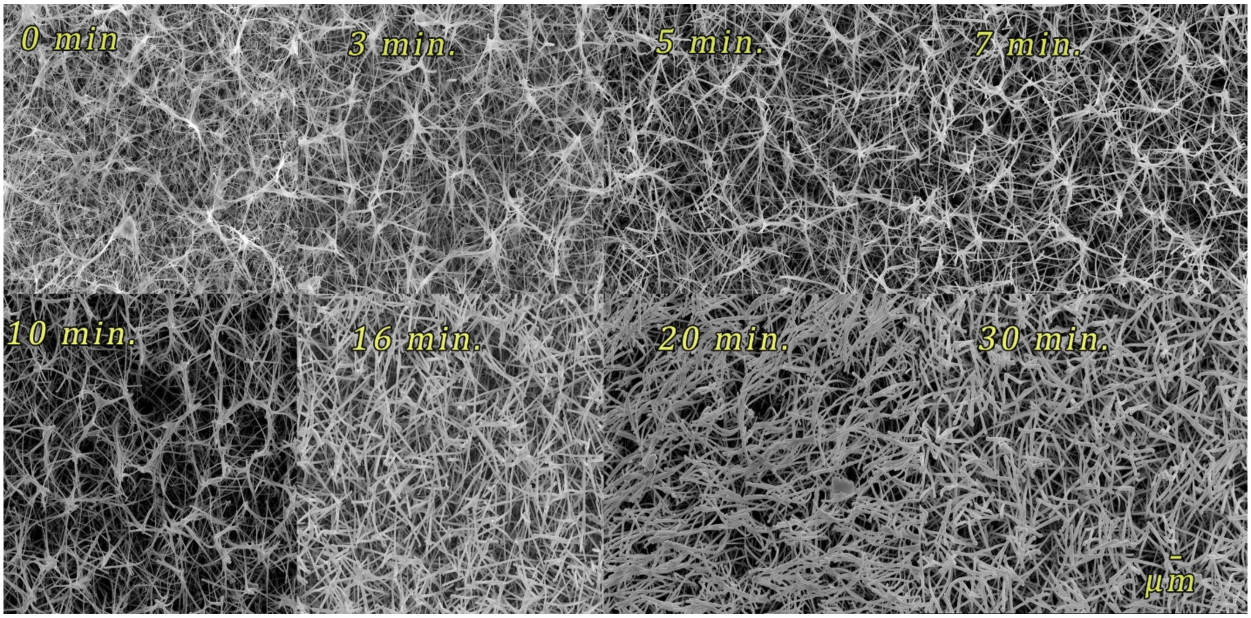

Figure S8: SEM images of samples after dipping in EtOH: SiNWs obtained at 500 °C by VLS deposition. Ag sputtering time: 3–30 min.

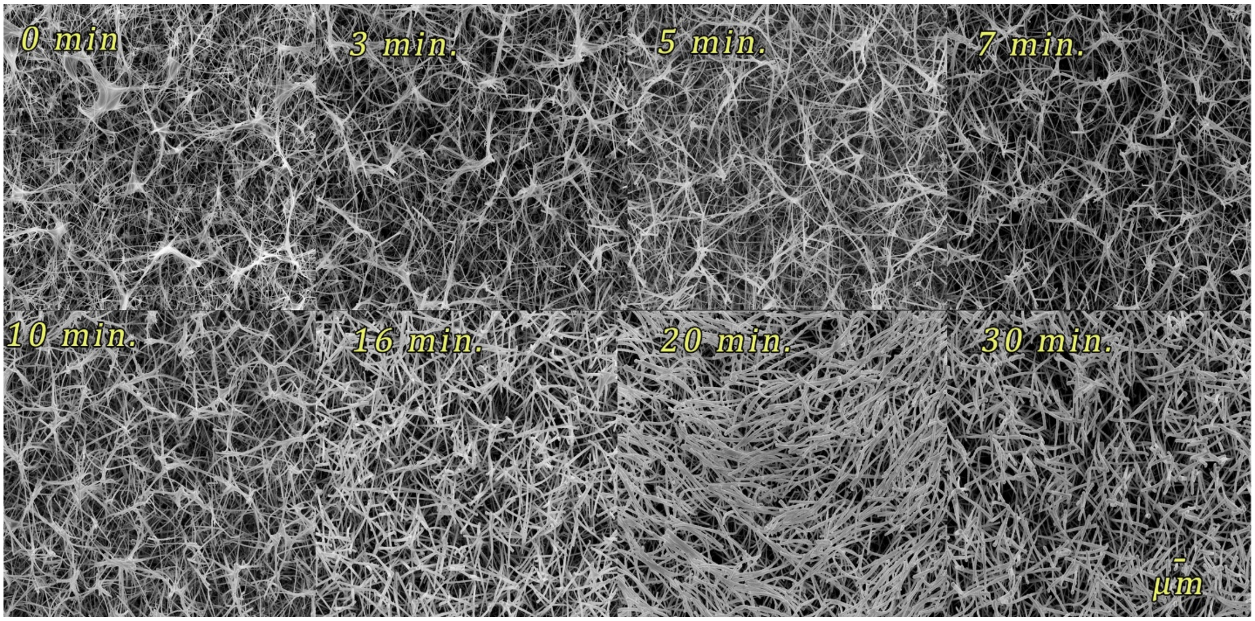

Figure S9: SEM images of samples after dipping in H_2O: SiNWs obtained at 500 °C by VLS deposition. Ag sputtering time: 3–30 min.

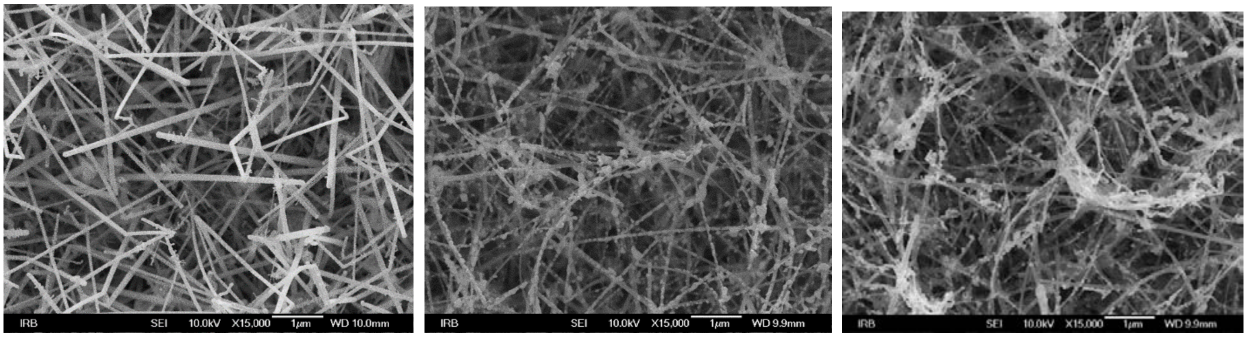

Figure S10: Comparison of samples: a) dry, b) after dipping in EtOH, c) after dipping in H_2O dip. Sputtering time for all samples was 5 min.

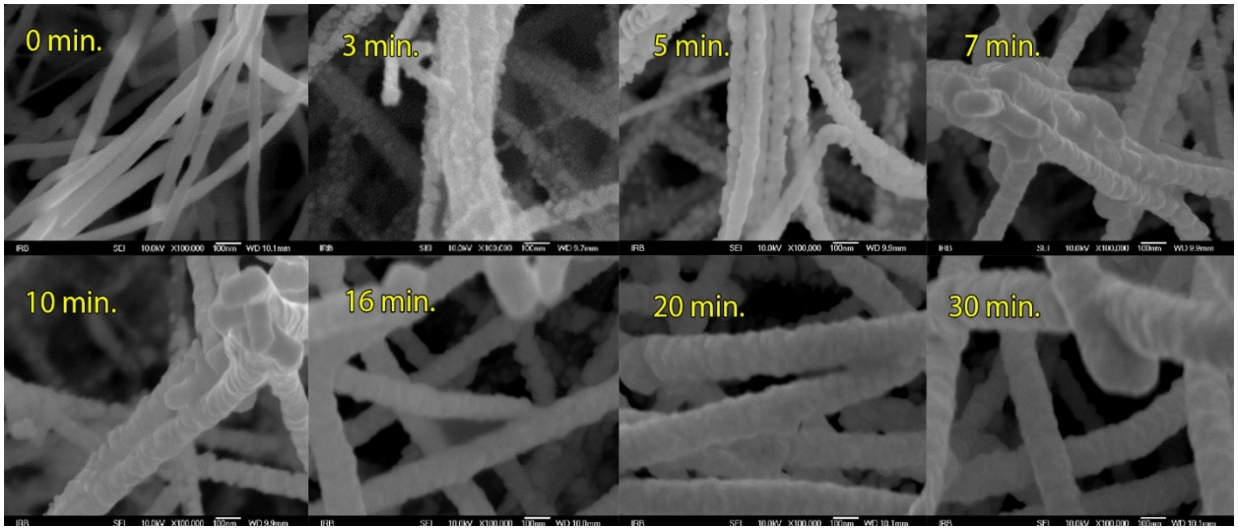

Figure S11: SEM images of SiNWs obtained by 500 °C VLS deposition with different Ag-sputtering times, after immersion in liquid. A strong capillary-force influence and SiNW-bundle formation in the samples sputtered for 3–10 min is observed.

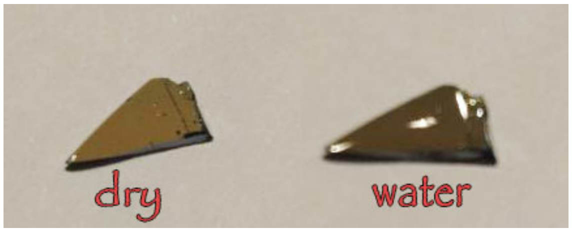

Figure S12: Dry and wet (7 µL H_2O drop) SiNWs substrate.

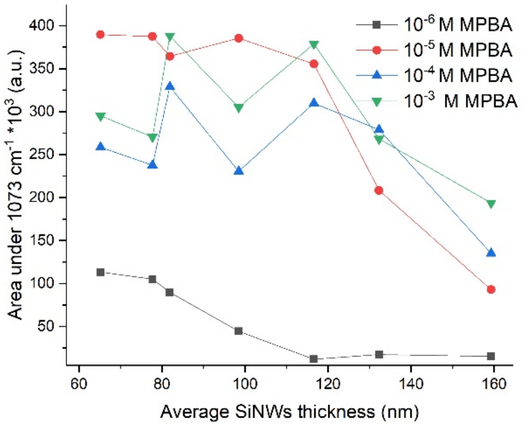

Figure S13: Average SERS values for different SiNWs thicknesses for 4-MPBA ethanol solutions.

SERS sensing of 4-MPBA

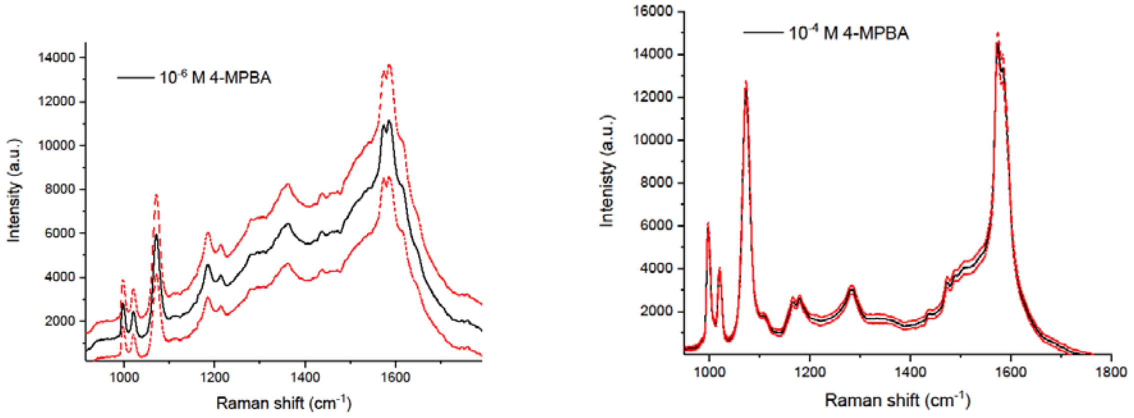

Figure S14: SiNW substrate after 5 min of Ag sputtering. 4-MPBA concentration: 10^{-6} M and 10^{-4} M. Mapping standard deviation (red curve) and the average SERS value (black curve). Mapping features: 100 points, 10 µm step, 1 scan/point, 10 s/scan.

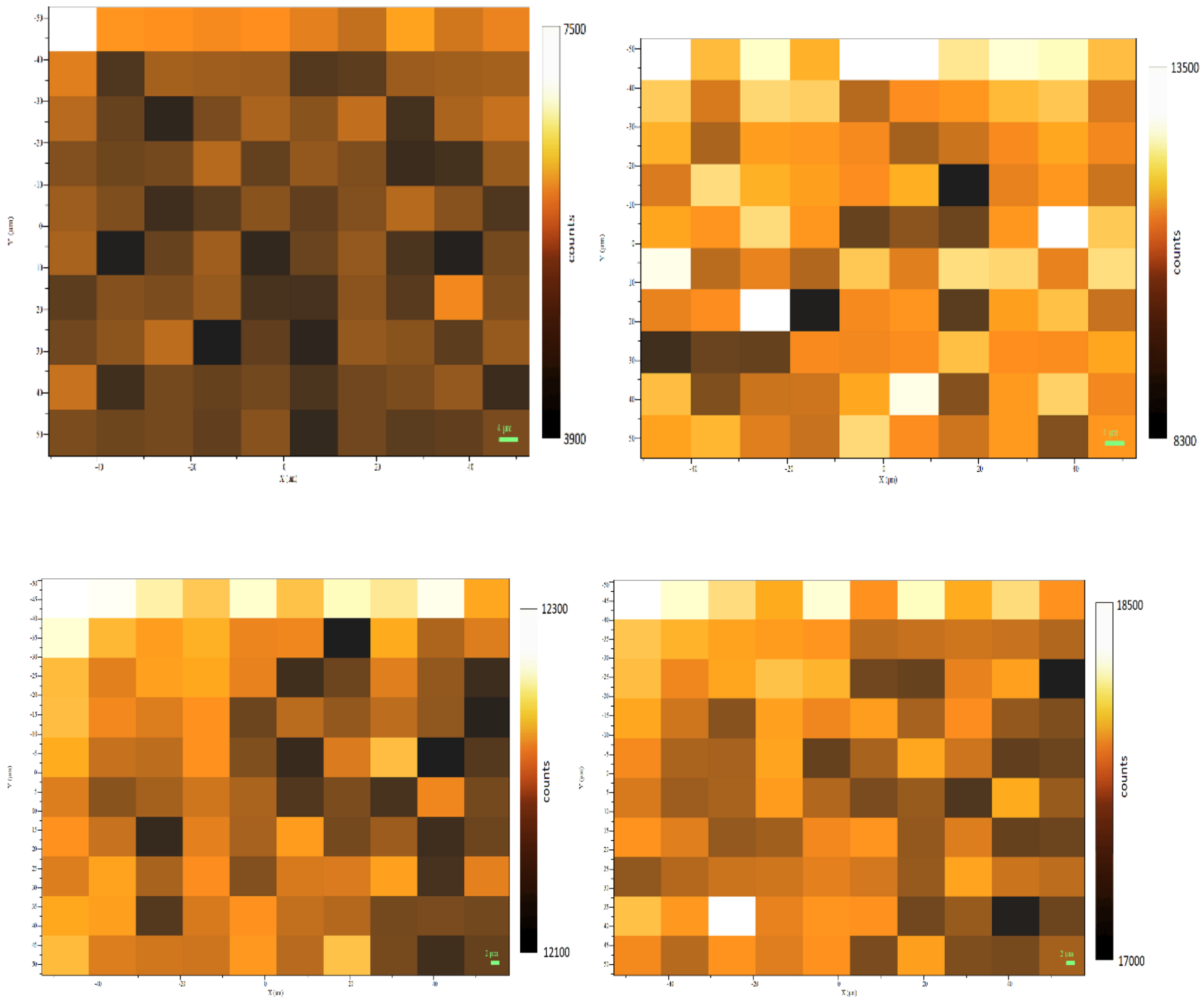

Figure S15: SERS mapping: a) 1075 cm^{-1} band, 10^{-6} M MPBA, 5 min Ag-sputtering time, b) 1574 cm^{-1} band, 10^{-6} M MPBA, 5 min Ag-sputtering time, c) 1075 cm^{-1} band, 10^{-4} M MPBA, 3 min Ag-sputtering time, d) 1574 cm^{-1} band, 10^{-4} M MPBA 3 min Ag-sputtering time.

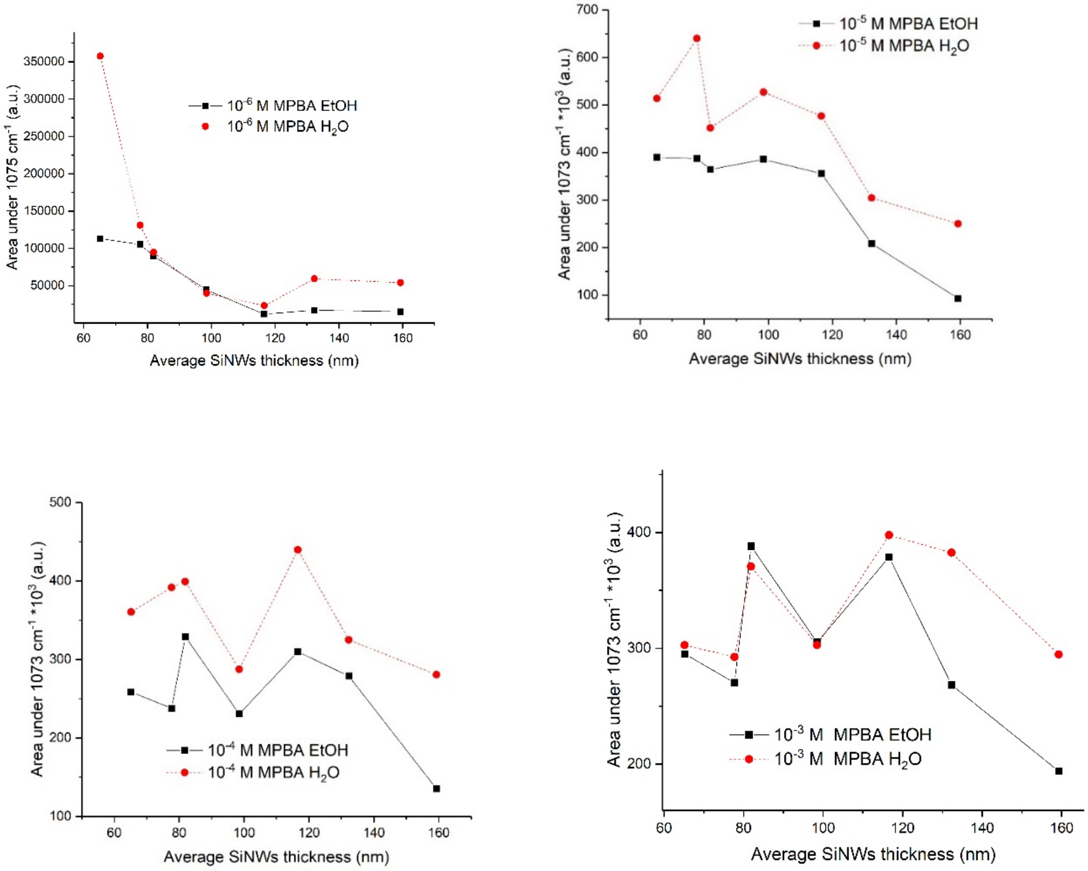

Figure S16: Integral SERS spectra intensities under the 1073 cm^{-1} band after immersion in EtOH or H_2O.

Lacunarity of SiNWs substrates

For the lacunarity calculation, we used ImageJ program utilizing only the same size (1875 × 1410 pixels or 8.3 × 6.2 μm) grey-scale SEM images. The program gave 12 logarithmic spectra as output from which the average value was taken. For example, the spectra for dry samples and different thickness values are shown in Figure S17. On the *x*-axis, the ε stands for: ε = ε(px)/SEM-figure dimension (px), where the "box size" is in pixels (px). The resolution is 8300 nm/1875 px = 4.4 nm/px and therefore the 532 nm laser-excitation wavelength corresponds to 532/4.4 = 120.9 px. This is the relevant sampling window size. Furthermore, this number should be divided by the SEM image size which is 1875 px, i.e., 120.9/1875 = 0.064484 which finally gives ln(0.064484) = −2.74. The values at −2.74 in Figure S9 are taken from all spectra for different SiNW thickness values and before and after H_2O immersion.

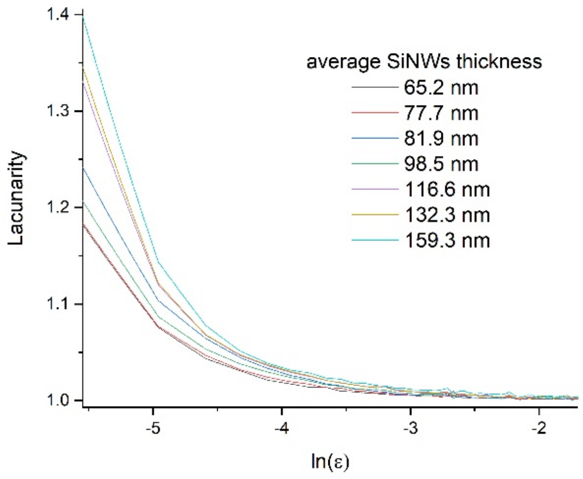

Figure S17: Lacunarity of dry SiNWs as a function of ε.

Comparison with commercially available SERS substrates

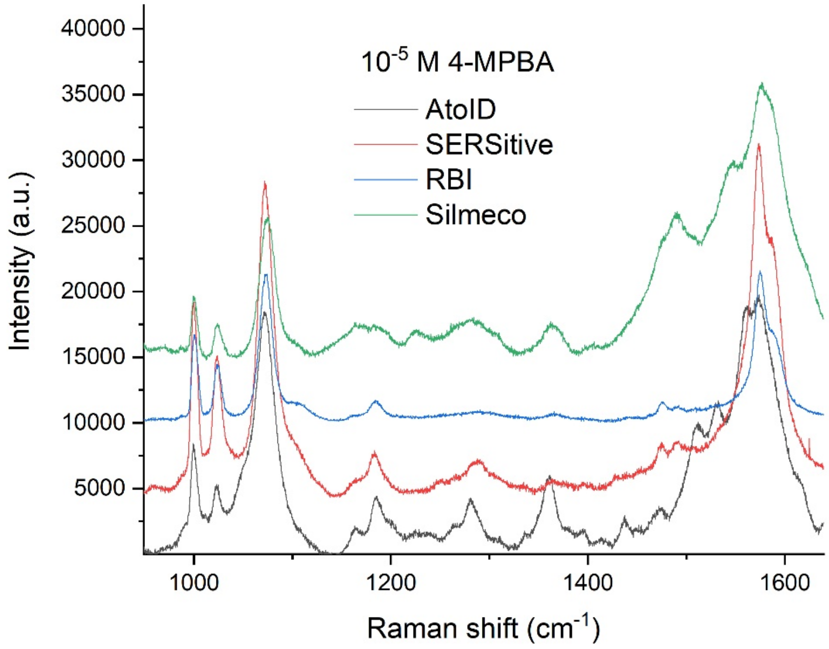

Figure S18: Comparison of the commercial SERS substrates and the synthesized sample (RBI) before immersion in water.

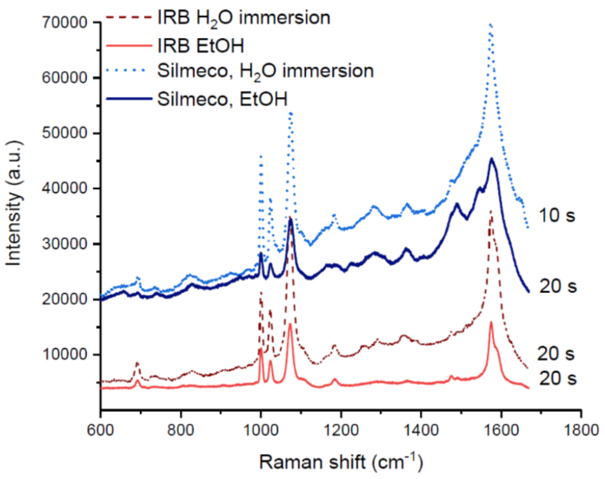

Figure S19: Comparison of Silmeco and IRB substrates for 10^{-5} M 4-MPBA.

References

1. Sun, F.; Ella-Menye, J.-R.; Galvan, D. D.; Bai, T.; Hung, H.-C.; Chou, Y.-N.; Zhang, P.; Jiang, S.; Yu, Q. *ACS Nano* **2015,** *9,* 2668–2676. doi:10.1021/nn506447k
2. Gebavi, H.; Ristić, D.; Baran, N.; Mikac, L.; Mohaček-Grošev, V.; Gotić, M.; Ivanda, M. *Silicon* **2018,** 1–7. doi:10.1007/s12633-018-9906-0
3. Sun, D.; Qi, G.; Xu, S.; Xu, W. *RSC Adv.* **2016,** *6,* 53800–53803. doi:10.1039/C6RA06223G
4. Sun, X.; Stagon, S.; Huang, H.; Chend, J.; Lei, Y. *RSC Adv.* **2014,** *4,* 23382. doi:10.1039/c4ra02423k
5. Xie, D.; Zhu, W. F.; Cheng, H.; Yao, Z. Y.; Li, M.; Zhao, Y. L. *Phys. Chem. Chem. Phys.* **2018,** *20,* 8881–8886. doi:10.1039/c7cp08478a
6. Pham, X.-H.; Shim, S.; Kim, T.-H.; Hahm, E.; Kim, H.-M.; Rho, W.-Y.; Jeong, D. H.; Lee, Y.-S.; Jun, B.-H. *BioChip J.* **2017,** *11,* 46–56. doi:10.1007/s13206-016-1107-6
7. Kiran, V.; Sampath, S. *ACS Appl. Mater. Interfaces* **2012,** *4,* 3818–3828. doi:10.1021/am300349k
8. Su, H.; Wang, Y.; Yu, Z.; Liu, Y.; Zhang, X.; Wang, X.; Sui, H.; Sun, C.; Zhao, B. *Spectrochim. Acta, Part A* **2017,** *185,* 336–342. doi:10.1016/j.saa.2017.05.068
9. Gao, P.; Weaver, M. J. *J. Phys. Chem.* **1985,** *89,* 5040–5046. doi:10.1021/j100269a031
